# Fine-tuning of lysine side chain modulates the activity of histone lysine methyltransferases

**DOI:** 10.1038/s41598-020-78331-0

**Published:** 2020-12-09

**Authors:** Abbas H. K. Al Temimi, Jona Merx, Christian J. van Noortwijk, Giordano Proietti, Romano Buijs, Paul B. White, Floris P. J. T. Rutjes, Thomas J. Boltje, Jasmin Mecinović

**Affiliations:** 1 Institute for Molecules and Materials, Radboud University, Heyendaalseweg 135, 6525 AJ Nijmegen, The Netherlands; 2 Department of Physics, Chemistry and Pharmacy, University of Southern Denmark, Campusvej 55, 5230 Odense, Denmark

**Keywords:** Transferases, Biocatalysis, Peptides, Post-translational modifications

## Abstract

Histone lysine methyltransferases (KMTs) play an important role in epigenetic gene regulation and have emerged as promising targets for drug discovery. However, the scope and limitation of KMT catalysis on substrates possessing substituted lysine side chains remain insufficiently explored. Here, we identify new unnatural lysine analogues as substrates for human methyltransferases SETD7, SETD8, G9a and GLP. Two synthetic amino acids that possess a subtle modification on the lysine side chain, namely oxygen at the γ position (K_O_, oxalysine) and nitrogen at the γ position (K_N_, azalysine) were incorporated into histone peptides and tested as KMTs substrates. Our results demonstrate that these lysine analogues are mono-, di-, and trimethylated to a different extent by trimethyltransferases G9a and GLP. In contrast to monomethyltransferase SETD7, SETD8 exhibits high specificity for both lysine analogues. These findings are important to understand the substrate scope of KMTs and to develop new chemical probes for biomedical applications.

## Introduction

The unstructured and flexible N-terminal histone tails are subject to a plethora of posttranslational modifications (PTMs), including methylation, acetylation, phosphorylation, ubiquitination, and many others^1^. These modifications are key regulators of the stability and activity of histones, biomolecular interactions, and the structure and function of human chromatin^2–4^. Histone PTMs have impact on various cellular processes, such as DNA repair, recombination and replication, and transcription^1^. Among histone PTMs, lysine methylation is linked with gene activation (H3K4, H3K36, and H3K27) and suppression (H3K9, H3K27, and H4K20), depending on the site and methylation states of the histone sequences^5,6^.

**Figure 1:**
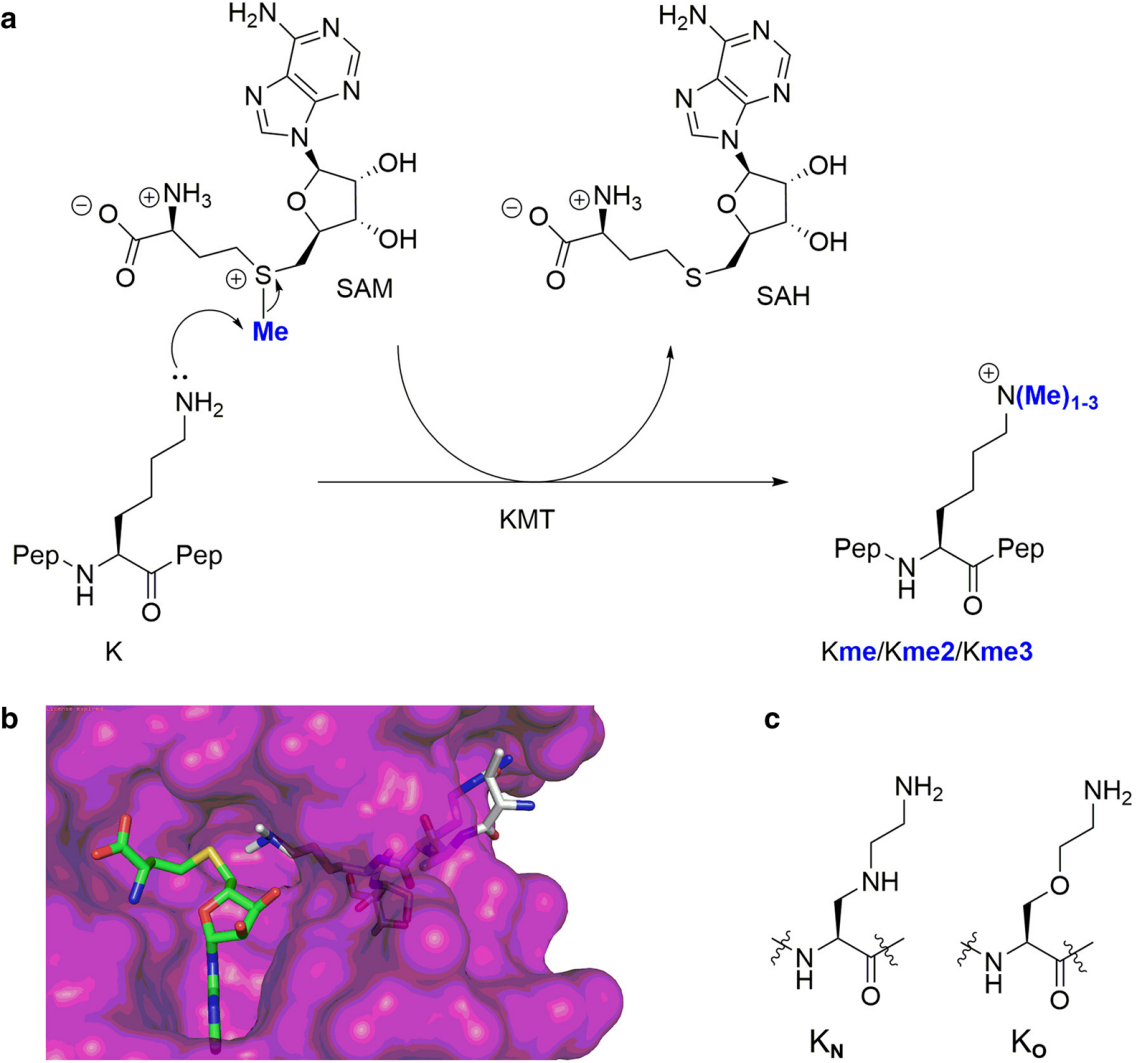
Histone lysine methylation. (**a**) Histone lysine methyltransferase (KMT)-catalyzed methylation of lysine in the presence of SAM cosubstrate. (**b**) View from a crystal structure of GLP complexed with the H3K9me2 histone peptide and SAH (PDB ID: 2RFI). (**c**) Lysine analogues K_N_ and K_O_ examined in the current study.

Lysine methylation on the N^ε^-amino group is catalyzed by histone lysine methyltransferases (KMTs), using *S*-adenosylmethionine (SAM) as the methylating agent. Methylated lysine residues exist in the form of monomethyllysine (Kme), dimethyllysine (Kme2), and trimethyllysine (Kme3) (Fig. 1a)^7,8^. Histone lysine methylation can be removed by lysine demethylases (KDMs), and is recognized by specific N^ε^-methyllysine binding reader proteins^1^. Unsurprisingly, defects in these epigenetic regulators are linked with various diseases, including, but not limited to, cancer^9^. Along with other enzymes involved in epigenetic gene regulation, KMTs represent promising drug targets in search for small molecules inhibitors^10,11^. Determined crystal structures of KMTs revealed that the histone peptide substrate and the SAM cosubstrate bind on opposite surfaces of the SET domain^12,13^. A proper orientation of lysine’s ^ε^NH_2_ group towards the electrophilic methyl group of SAM results in the formation of the methylated lysine and *S*-adenosylhomocysteine (SAH) via classic S_N_2 displacement (Fig. 1a)^14,15^. Generally, the lysine substrate occupies a narrow hydrophobic channel that crosses the core of the catalytic domain of KMTs and links to the SAM binding site (Fig. 1b)^16^. The final methylation state on the ^ε^NH_2_ group appears to be dependent on the presence of Tyr/Phe residues in the active site of KMTs^13^.

Recent molecular studies have shown that KMTs have a limited substrate scope, accepting only the simplest lysine analogues as substrates^17–23^ and SAM analogues as cosubstrates^24–31^. Enzymatic evaluations of unnatural lysine analogues as substrates for human KMTs demonstrated that KMTs are non-promiscuous regarding the chain length of lysine residue^22^, and the lysine stereochemistry^19^. The main chain of lysine in histones was found to be crucial for the catalytic activity of KMTs^17^, while the conformational freedom of the lysine side chain appear to play an important role in efficient KMT catalysis^23^. Recent studies revealed that the introduction of heteroatoms in the lysine side chain at the γ-position (by S)^18^ and the ε-position (by O and N) lead to excellent KMT substrates^21^. Enzymatic assays and computational studies pinpointed that the N^ε^-lysine functionality is exclusively required for the biocatalytic potential of KMTs and that the terminal amino group of lysine in histone peptides cannot be replaced by non-amino nucleophiles^21^.

To further investigate the role of lysine modification on the catalytic conversion and selectivity towards KMTs, we aimed to fine-tune the lysine side chain. Following our recent observations that replacement of the γ-methylene group by sulfur resulted in efficient KMT substrates^18^, we hypothesized that a more pronounced variation of the chain length and nucleophilicity, by introducing other heteroatoms at the γ-position, may lead to modulation of the substrate specificity or activity of KMTs to an extent that different methylation states of products could be achieved. We therefore prepared histone peptides containing lysine analogues possessing an NH (K_N_) and O (K_O_) at the γ-position to study the promiscuity of KMTs and delineate their selectivity (Fig. 1c).

## Results and discussion

**Figure 2:**
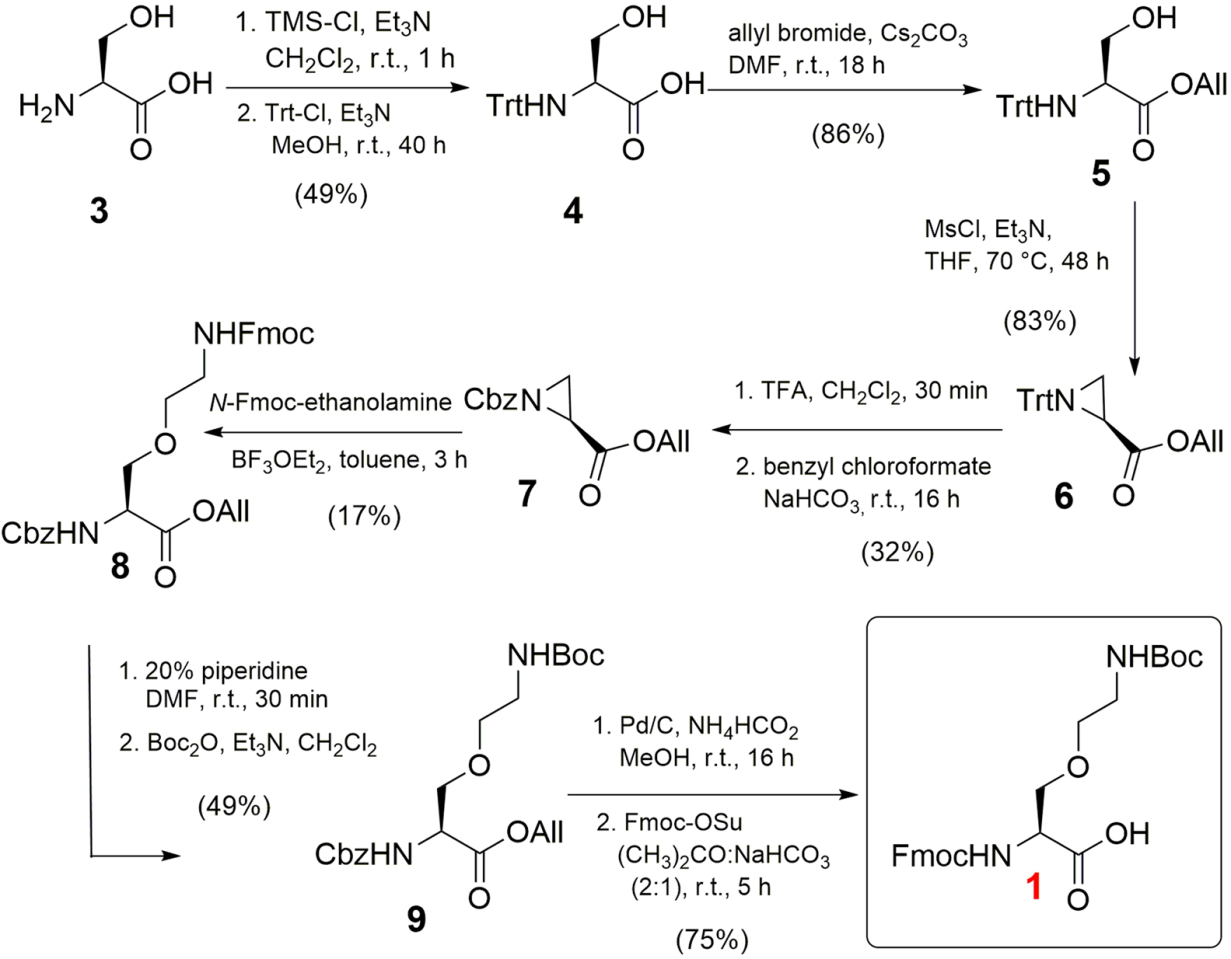
Synthesis of Fmoc-K_O_(Boc)-OH (**1**).

The synthesis of γ-oxalysine (K_O_) was envisioned by Lewis acid-catalyzed nucleophilic opening of serine-derived aziridine **8** by *N-*Fmoc-ethanolamine, yielding the desired building block **1** after protecting group modifications. This particular analogue has not been synthesized before, albeit that a similar threonine-derived building block was described by Gellman and co-workers, and by Vederas et al.^32,33^. Initially, we first protected the carboxylic acid of l-serine as an allyl ester followed by tritylation of the α-amino functionality. However, the initial Brønsted-catalyzed allylation under reflux conditions to produce compound **5** resulted in disappointingly low yields, mainly due to self-condensation of serine under the employed conditions. Alternatively, the first protection of the amino group of serine **3** with a trityl group to produce **4**, and subsequent allylation afforded **5** in 42% yield over two steps, comparable to an earlier literature report^34^. The protected serine **5** was treated with MsCl under reflux conditions for 48 h to cleanly afford aziridine **6** in good yield comparable to the Thr analogue^32,33^. The trityl group was replaced with a benzyl carbamate (Cbz) in two steps to allow for a Lewis acid-catalyzed nucleophilic ring opening. This step was successfully achieved by employing BF_3_·Et_2_O (0.5 equiv) under reflux conditions for 2 h in toluene, yielding **8**. A one-pot replacement of the Fmoc group of **8** into Boc gave the penultimate protected amino acid **9**. To allow for use in standard solid phase peptide synthesis (SPPS), both the allyl and Cbz protecting groups were removed by H_2_ and Pd/C. A subsequent one-pot protection of the amine with Fmoc-OSu was performed to obtain the novel Fmoc-K_O_(Boc)-OH (**1**) in 75% yield (Fig. 2).

**Figure 3:**
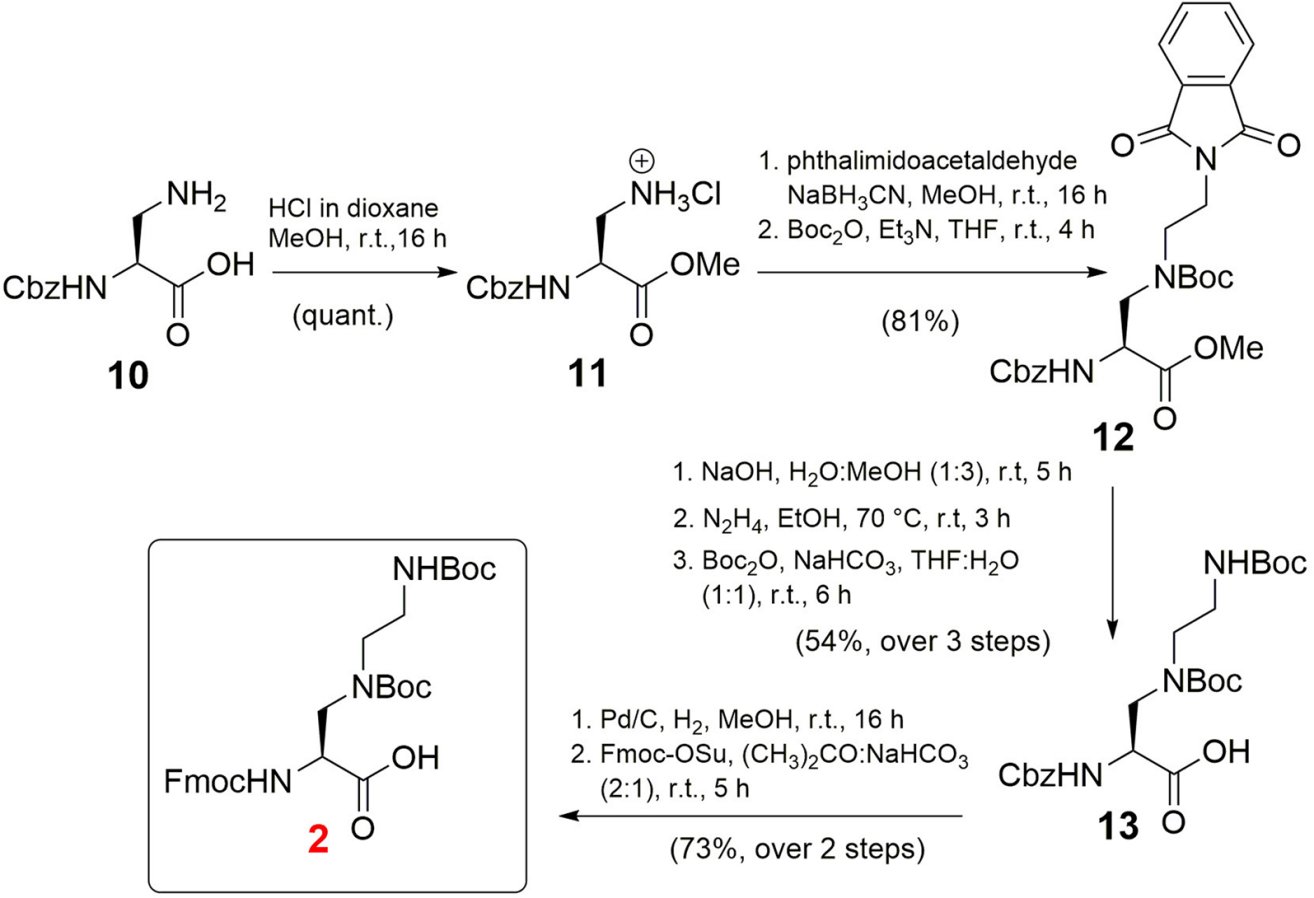
Synthesis of Fmoc-Kn(Boc)_2_-OH (**2**).

Next, the synthesis of Fmoc/Boc-protected γ-azalysine (K_N_) **2** was achieved in 8 steps starting from Cbz-Dab-OH **10** (Fig. 3). The synthesis of this building block has been previously reported^35^. However, we designed a new synthetic route to produce this building block employing the readily available phthalimidoacetaldehyde thereby improving the overall yield (31%, 8 total steps) compared to a synthesis reported by Chhabra et al*.* (10%, 8 steps from **10**)^35^. The synthesis started by protecting **10** as a methyl ester (**11**) in quantitative yield. After reductive amination of amine **11** with phthalimidoacetaldehyde, followed by protection of the secondary amine with a Boc group, intermediate **12** was obtained in a yield of 81%. Initially, selective deprotection of the phthalimide group in **12** was attempted with hydrazine, however, under the employed conditions the methyl ester was mainly converted into the corresponding acylhydrazide. To circumvent the formation of this byproduct, the ester was first hydrolysed under basic conditions. Subsequent phthalimide deprotection with hydrazine and protection of the resulting primary amine in the presence of Boc anhydride resulted in amino acid **13** in 54% yield over 3 steps. Final one-pot hydrogenolysis of the Cbz group, followed by Fmoc protection afforded the desired building block Fmoc-K_N_(Boc)_2_-OH (**2**) in 73% yield (Fig. 3).

**Figure 4:**
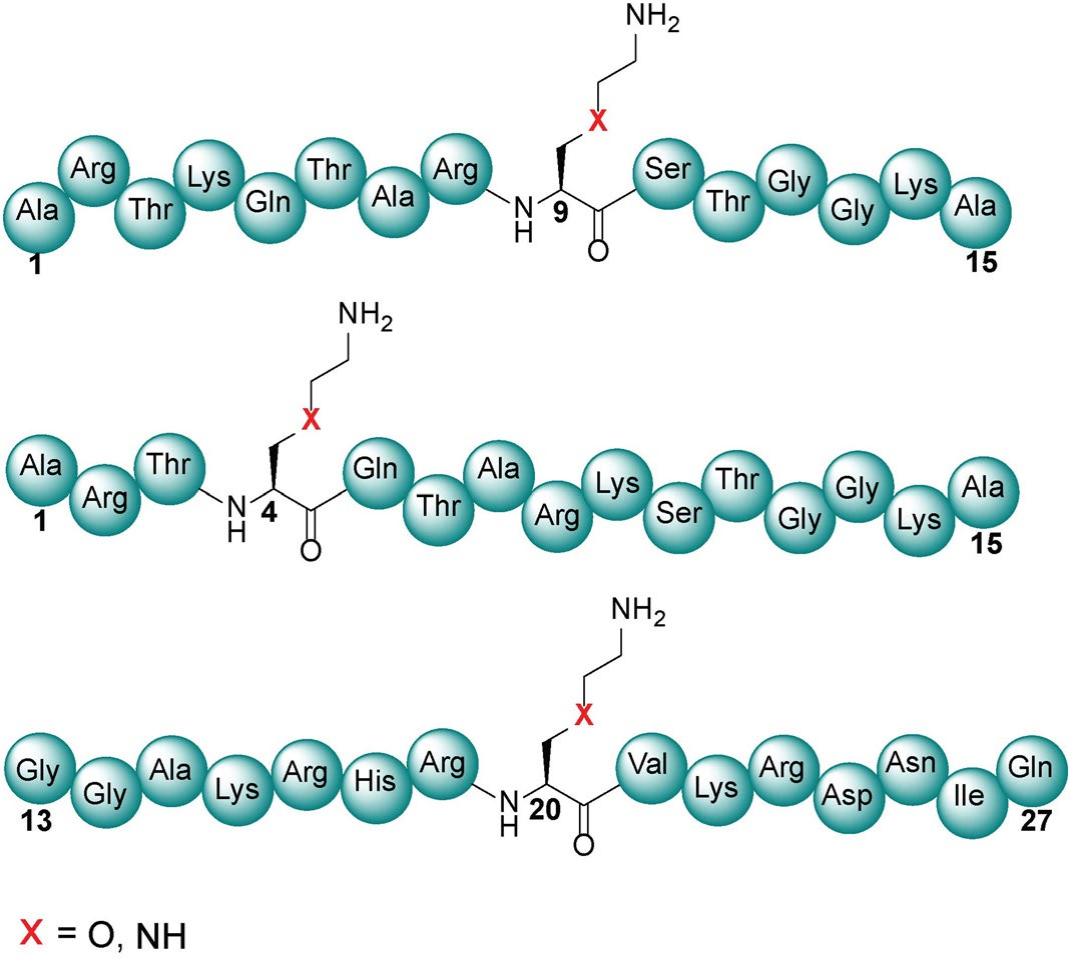
Representative structures of histone H3K9, H3K4, and H4K20 peptides where the natural lysine has been replaced by both γ-oxalysine and γ-azalysine at specific positions for KMT catalysis.

With building blocks **1** and **2** in hand, we prepared histone peptide fragments containing the modified lysines for evaluation with human KMTs. To this end, six new peptides (Fig. 4) and three native peptides were synthesized by SPPS using Fmoc/*t*-butyl chemistry (Supplementary Schemes S1–S3; Supplementary Figs. S1–S3). The prepared histone peptides contained the N-terminal 15-residues of H3 (ARTKQTARK^9^STGGKA) in which K9 was replaced with either of the two analogues for evaluation with GLP and G9a. Additionally, the two analogues were also inserted at position 4 (ARTK^4^QTARKSTGGKA) to be examined with SETD7. To further expand the enzyme scope, we also prepared peptide fragment 13–27 of H4 (GGAKRHRK^20^VLRDNIQ), in which K20 was replaced with K_O_ and K_N_ for evaluation with SETD8. All synthetic histone peptides were produced in high purity using RP-HPLC; their purity was confirmed by analytical HPLC and their identity by LC–MS and MALDI-TOF MS (Supplementary Figs. S4–S6).

**Figure 5:**
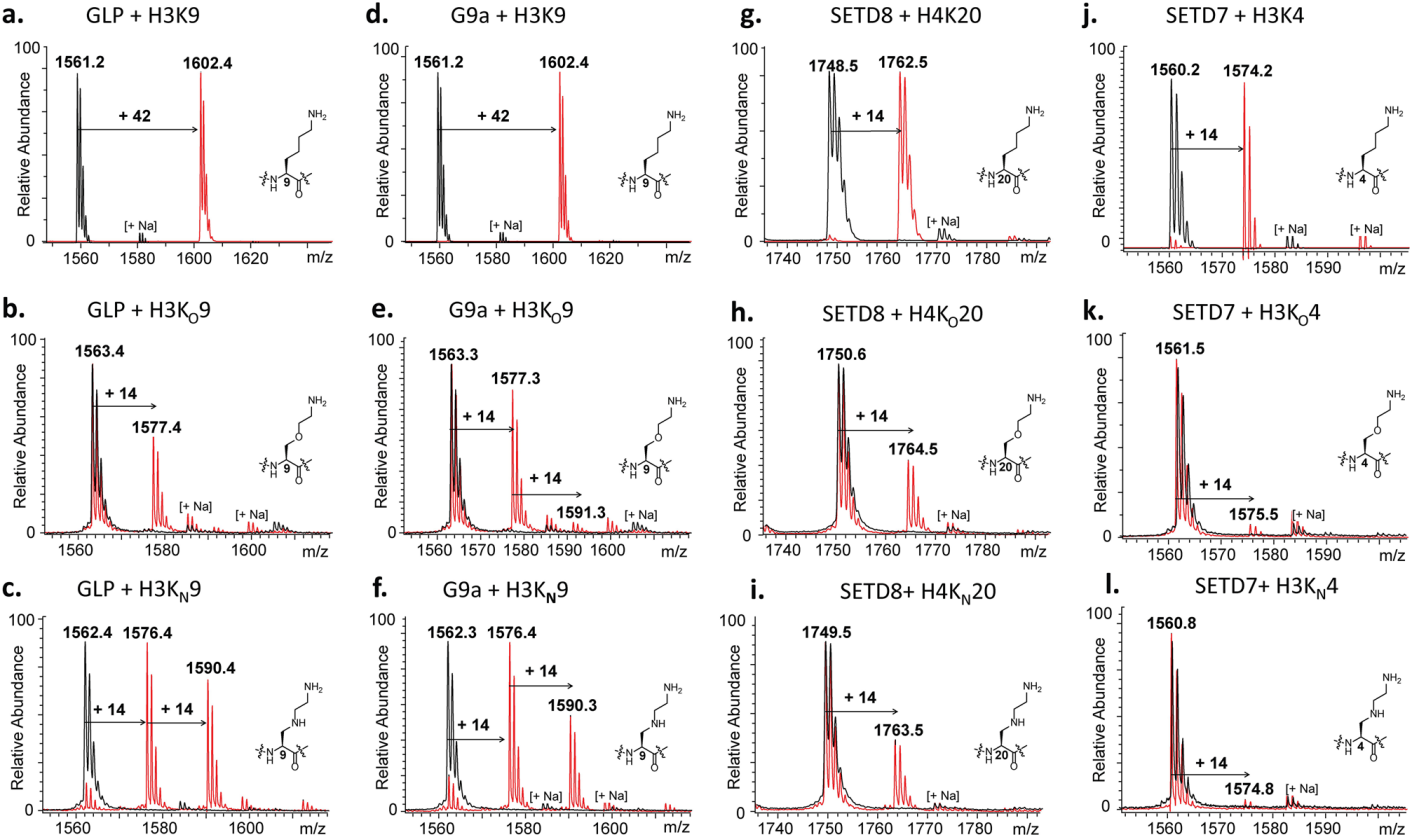
KMT-catalyzed methylation of lysine and its analogues that possess O and NH functionalities at the γ-position. MALDI-TOF MS data showing methylation of histone peptides in the presence of GLP with (**a**) H3K9, (**b**) H3K_O_9, (**c**) H3K_N_9; and G9a with (**d**) H3K9, (**e**) H3K_O_9, (**f**) H3K_N_9; and SETD8 with (**g**) H4K20, (**h**) H4K_O_20, (**i**) H4K_N_20; and SETD7 with (**j**) H3K4, (**k**) H3K_O_4, (**l**) H3K_N_4. Red spectra show 1 h reactions at 37 °C including KMTs (2 µM), histone peptide (100 µM) and SAM (500 µM for GLP/G9a and 200 µM for SETD8/SED7) and black spectra the no-enzyme controls.

Histone peptides bearing the lysine analogues K_O_ and K_N_ were examined as substrates for the recombinantly expressed human KMTs using MALDI-TOF MS assays as a direct method to monitor histone methylation. We tested whether K_O_ and K_N_ can be methylated by di- and trimethyltransferases GLP and G9a, and monomethyltransferases SETD7 and SETD8. Samples containing the human KMT enzyme (2 µM), histone peptide (100 µM), and SAM (500 µM with GLP and G9a; 200 µM with SETD8 and SETD7) in Tris buffer (50 mM, pH 8.0) were incubated for 1 h at 37 °C. Following established enzymatic conditions^17–19,21,22^, all lysine-containing histone peptides were efficiently methylated by the four KMTs (Fig. 5a,d,g,j, respectively). Then the assessment of H3K_O_9 and H3K_N_9 peptides with GLP and G9a was carried out. Unlike trimethylation of lysine normally observed under the assay conditions in vitro, both enzymes catalyzed methylation of the H3K_O_9 peptide to produce monomethylated H3K_O_9me, as the characteristic methyl mass shift of + 14 Da was clearly detectable in comparison with the unmethylated peptide (Fig. 5b,e). In the G9a assay, traces (< 5%) of the dimethylated species H3K_O_9me2 were also observed. MALDI-MS assays with H3K_N_9 showed that both enzymes catalyzed the formation of monomethylated H3K_N_9me and dimethylated H3K_N_9me2 (Fig. 5c,f). Omission of GLP/G9a resulted in no observable methylation, indicating an enzymatic process (black spectra in Fig. 5). We further analyzed the product distribution of these two peptide substrates with G9a and GLP throughout the time course of methylation. The MALDI-TOF MS assignment revealed an increase in degree of methylation with time (Supplementary Figs. S7, S8). At high concentrations of enzyme (10 µM) and SAM (1 mM), GLP and G9a provided predominantly dimethylated H3K_O_9me2 and some degree of trimethylated H3K_O_9me3; the degree of trimethylation increased upon prolonged incubation (Supplementary Figs. S9–S13). Similar results were observed with both enzymes upon screening the H3K_N_9 peptide at high concentration, as the trimethylated product H3K_N_9me3 was observed upon prolonged incubation (Supplementary Figs. S9–S13). These data indicate that the G9a/GLP-catalyzed first methylation reaction is the fastest, whereas the second and third methylations are comparatively slower, producing increasing amounts of higher methylation states products over time. It is worth noting that introduction of γ-oxalysine and γ-azalysine does not lead to complete alteration of the substrate specificity, but rather to modulation of activity by KMTs. Absence of the enzyme resulted in no observable methylation reaction (black spectra in the Supplementary Figs. S12, S13).

Having established that GLP and G9a catalyzed efficient methylations of H3K_O_9 and H3K_N_9, we sought to investigate K_O_ and K_N_ with other members of the KMTs, monomethyltransferases SETD8 and SETD7. Applying the standard assay conditions, SETD8 notably catalyzed the monomethylation of H4K_O_20 and H4K_N_20 (Fig. 5h,i). Nearly complete monomethylation was observed with an increased concentration of SETD8 (10 µM) and SAM (1 mM) after 1 h at 37 °C (Supplementary Fig. S14). On the other hand, examining these two analogues in the presence of SETD7 resulted in only traces (< 5%) of monomethylated species (Fig. 5k,l). The lack of methylation of these two analogues with SETD7 indicated a selectivity between KMTs. Increased amounts of SETD7 (10 µM) and SAM (1 mM), and prolonged incubation times (3 h) did not lead to formation of the monomethylated products either (only traces of H3K_O_4me and H3K_N_4me observed, Supplementary Fig. S15). Overall, the results show that histone lysine methyltransferases possess different activities towards lysine and simple lysine analogues; monomethyltransferase SETD7 is more restrictive towards K_O_ and K_N_ compared to SETD8 and trimethyltransferases GLP and G9a.

To determine the substrate specificity of SETD8 towards the two histone peptides bearing the unnatural lysine analogues K_O_ and K_N_, a kinetic investigation was carried out employing a MALDI-TOF MS enzymatic assay^17,18,21^. KMT-catalyzed methylation of the oxalysine- and azalysine-containing histone peptides was characterized by slower substrate conversion rates (k_cat_) compared to their respective natural sequences, a result that we attribute to their poorer nucleophilic character due to the electron-withdrawing properties of the O and NH moieties. Furthermore, a highly hydrophobic channel of the methyltransferases is poorly tailored for significant electrostatic changes in the hydrophobic nature of the lysine side chain, likely leading to a larger penalty for desolvation of γ-azalysine and γ-oxalysine. Combined with the altered K_m_ values for H4K_O_20 and H4K_N_20, this translated into a generally slightly worse catalytic efficiency for these two lysine analogues. Taken together, H4K_O_20 and H4K_N_20 were found as efficient substrates for SETD8 catalysis, with 2.1- and 2.8-fold decreases in k_cat_/K_m_ values, respectively (Table 1; Supplementary Fig. S16). Comparisons of kinetics data for H4K20, H4K_O_20, H4K_N_20 with the highly related analogue H4K_C_20 (ref.^18^) shows that SETD8 very well tolerates subtle modifications at the γ position of the side chain, in the order H4K20 > H4K_C_20 > H4K_N_20 > H4K_O_20. Following these trends, we anticipate that the high substrate specificity of SETD7 for H3K4 over analogues is a result of high K_m_ values for the latter^18^; more detailed kinetics analyses on H3K_N_4 and H3K_O_4 were therefore not possible, as very low levels of methylation were detected.

**Table 1:** Kinetics parameters for SETD8-catalyzed methylation of H4K20, H4K_O_20 and H4K_N_20.

H4 peptide	K_m_ (μM)	k_cat_ (min^−1^)	k_cat_/K_m_ (mM^−1^ min^−1^)
H4K20	103.9 ± 25.3	0.54 ± 0.11	5.21
H4K_O_20	172.0 ± 54.7	0.32 ± 0.11	1.86
H4K_N_20	59.5 ± 9.5	0.15 ± 0.01	2.50

Residual activity assays monitoring the KMT-catalyzed methylation of the histone peptides were then carried out, aimed at providing competitive data of these two substrates as compared to the 14-mer H3K9 peptide for the active site of G9a/GLP, and to determine whether the two lysine analogues inhibit G9a- and GLP-catalyzed methylation of H3K9. Both substrates appear to be very poor inhibitors of G9a and GLP (IC_50_ > 100 µM), indicating that the H3K9 peptide outcompetes H3K_O_9 and H3K_N_9 peptides for binding in the active site of G9a/GLP (Supplementary Fig. S17). An examination of 15-mer H3K_O_4 and H3K_N_4 peptides as inhibitors of SETD7 using the 14-mer H3K4 substrate revealed that H3K_O_4 exhibits weak inhibition activity (IC_50_ = 38.9 µM), whereas H3K_N_4 appears to be inactive (IC_50_ > 100 µM) (Supplementary Figs. S18, S19).

**Figure 6:**
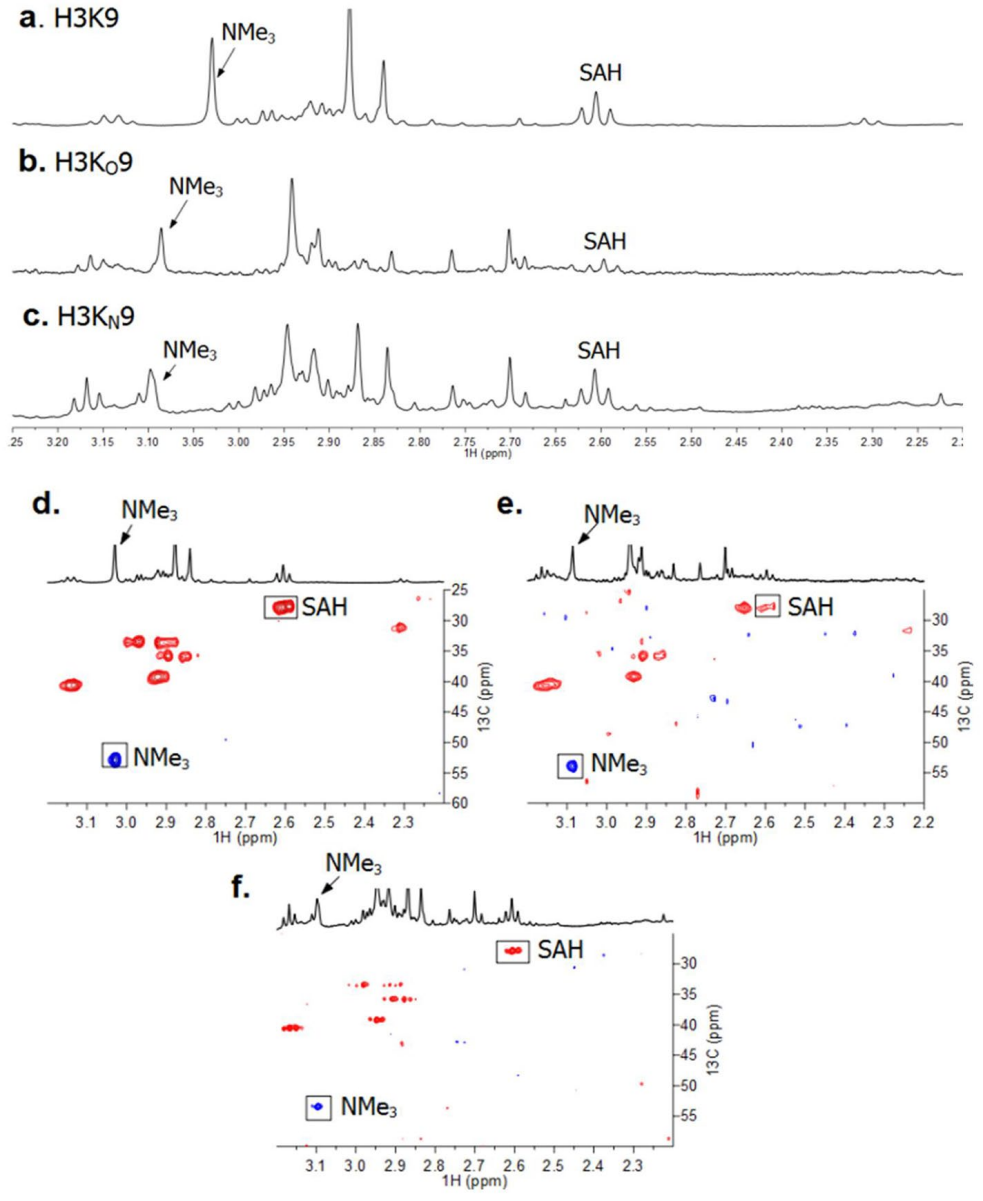
NMR analyses of GLP-catalyzed methylation reactions. ^1^H NMR of (**a**) H3K9; (**b**) H3K_O_9; and (**c**) H3K_N_9 spectra revealing methylation of histone peptides in the presence of GLP and SAM. ^1^H-^13^C HSQC data of (**d**) H3K9; (**e**) H3K_O_9; and (**f**) H3K_N_9 data with the assignment of cross-peaks. Correlations corresponding to the methylated lysine analogues are highlighted.

To further validate the role of the lysine analogues as KMT substrates, we investigated the detection of GLP-catalyzed methylation of H3K_O_9 and H3K_N_9 by ^1^H NMR and ^1^H-^13^C HSQC measurements. Before starting the enzymatic NMR studies, these histone peptides were analyzed by ^1^H NMR and ^1^H-^13^C HSQC (Supplementary Figs. S20, S21). The enzymatic NMR sample contained GLP (8 µM), the H3K_O_9 or H3K_N_9 peptide (400 µM) and SAM (2 mM) in Tris-*d*_11_ buffer at *p*D 8.0. After 1 h incubation at 37 °C, the samples were transferred to NMR tubes and then subjected to ^1^H NMR analyses. GLP-catalyzed methylation of H3K9 resulted in a signal of H3K9me3 at 3.03 ppm (^13^C 53.0 ppm, NMe_3_) as shown in Fig. 6a,d, respectively, in line with recent work^21,36^. A concomitant conversion of SAM to SAH was evidenced by the ^1^H NMR resonance at 2.62 ppm (corresponding to the methylene protons of SAH-CH_2_γ) for lysine and both lysine analogues (Fig. 6). H3K_O_9 and H3K_N_9 substrates showed the appearance of new singlet resonances at 3.09 ppm and 3.10 ppm, respectively (Fig. 6b,c). Subsequent ^1^H-^13^C HSQC measurements indicated that these signals were methyl groups of NMe_3_ with correlated carbon resonance at 53.6 ppm and 53.1, respectively (Fig. 6e,f). It is noteworthy that trimethylation of lysine analogues as observed by NMR occurs at high concentration of GLP, in line with MALDI-TOF data using high concentrations of GLP/G9a.

## Conclusion

Overall, our findings demonstrate that human KMTs in general have an ability to catalyze methylation of γ-azalysine and γ-oxalysine containing histones. Despite subtle chemical differences between lysine and its two simple analogues that differ in the γ position of the side chain, enzyme assays showed that different degrees of KMT-catalyzed methylation are observed. Methyltransferases G9a and GLP that catalyzed a complete trimethylation of H3K9 in vitro, predominantly catalyzed monomethylation of H3K_O_9, and mono- and dimethylation of H3K_N_9 at same conditions, demonstrating the fine-tuning of the lysine side chain leads to different methylation states. In addition, although SETD8 showed catalytic activity towards H4K_O_20 and H4K_N_20, the conversion of H3K_O_4 and H3K_N_4 by SETD7 was significantly reduced. These results highlight differences in activity of the human KMTs for methylation of simplest lysine analogues. Together with recent examinations of lysine analogues as substrates for KMTs, results presented here importantly contribute to a better understanding of biocatalysis of histone lysine methyltransferases that play essential roles in human health and disease.

## Methods

### Expression and purification of KMTs

Human KMTs enzymes were expressed and purified as previously described^36–38^. The methyltransferase plasmid (SETD8 residues 186–352, SETD7 residues 1–366, GLP residues 951–1235, G9a residues 913–1193) transformed into *Escherichia coli* Rosetta BL21 (DE3)pLysS cells. Cultures were grown at 37 °C in LB media containing kanamycin and chloramphenicol. Cells were grown to an OD_600_ of 0.5–0.6 (approximately 2.5–3 h), at which point they induced by isopropyl β-D-1-thiogalactopyranoside (IPTG) and they were transferred to a temperature of 16 °C overnight. After letting the cells grow at this temperature, they were then harvested and lysed by sonication. The lysate was centrifuged at high-speed to remove unbroken cells. The supernatant was then centrifuged to further clean the lysate. Purification of the N-terminally his6-tagged KMTs was performed using Ni–NTA affinity chromatography column, which was prewashed with lysis buffer. Target KMT enzyme was then eluted using a linear gradient concentration of imidazole. The elute was then concentrated with centrifugal concentrators (Millipore). All KMT enzymes were further purified by gel filtration on a Superdex 75 column (GE Healthcare) on an AKTA system. Purified proteins were concentrated employing Amicon Ultra Centrifugal Filter Units (Millipore) with suitable molecular weight cutoffs. Proteins concentrations were determined using the Nanodrop DeNovix DS-11 spectrophotometer and the purity was monitored by SDS-PAGE on a 4–15% gradient polyacrylamide gel (Bio-Rad).

### MALDI-TOF MS assays

Histone methyltransferase assay was carried out as described in 30 µL final total volume^17–19,21,22^. In brief, purified KMTs enzymes (2 µM of each enzyme per each reaction) were incubated with 100 µM of purified histone peptides in the methyltransferase assay buffer Tris–HCl (pH 8.0) and methyl donor SAM (500 µM with GLP and G9a; 200 µM with SETD8 and SETD7) for 1 h at 37 °C. 3 µL of reaction mixture was quenched with an equal amount of MeOH (1:1 ratio). Then, 3 µL of this mixture was added to an equal amount volume of a standard α-cyano-4-hydroxycinnamic acid matrix solution for spotting on a MALDI plate (Stainless ground steel 96/12 Bruker-Daltonik, Bremen-Germany). The mass spectra of peptides were analyzed by MALDI-TOF MS.

### Enzyme kinetics

Kinetics studies were performed as described^17,18,21^. A solution of histone peptide (0–300 μM), was added to a solution of SAM (116 μM) in assay buffer (50 mM Tris, pH 8.0) at room temperature (final volume of 100 μL). The reaction was then initiated by the addition of SETD8 (2 µM) and shaken for 10 min, and quenched by addition of methanol:water (1:1). All experiments were carried out in replicates. Kinetics values were extrapolated by plotting initial reaction velocities against peptide concentrations, utilizing GraphPad Prism 5.

### NMR activity assays

NMR spectroscopy was carried out as described^21^. NMR spectra were recorded using a Bruker AVANCE III (500 MHz ^1^H, 125 MHz ^13^C) spectrometer equipped with a Prodigy BB cryoprobe. All samples were prepared in Eppendorf vials (1.5 mL volume) before being transferred to NMR tubes. The D_2_O solvent signal was used as internal lock signal and any residual HOD signal was suppressed. Chemical shifts are reported relative to the solvent water resonance (4.7 ppm).

### Synthesis

#### 2-(9-Fluorenylmethoxycarbonylamino)ethanol^33,39^

Ethanolamine (1.98 mL, 32.8 mmol) was dissolved in 2:1 acetone:sat. aq. NaHCO_3_ (300 mL). Fmoc-OSu (11.04 g, 32.7 mmol) was added and the mixture was stirred at room temperature for 2 h. The acetone was removed *in vacuo*, and the residue was dissolved in water (200 mL) and EtOAc (200 mL). The mixture was acidified with 1 M HCl and the layers were separated. The organic layer was washed with 1 M HCl (3 × 200 mL), sat. aq. NaHCO_3_ (200 mL) and brine (200 mL). The combined organic phases were dried over Na_2_SO_4_ and the solvent was removed *in vacuo* to yield a white solid (8.94 g, 97%). Rf 0.56 (1:9 MeOH:DCM); ^1^H NMR (500 MHz, Chloroform-*d*) δ 7.76 (dt, *J* = 7.6, 0.9 Hz, 2H), 7.59 (dd, *J* = 7.5, 1.0 Hz, 2H), 7.40 (tt, *J* = 7.5, 0.9 Hz, 2H), 7.31 (td, *J* = 7.4, 1.1 Hz, 2H), 5.17 (s, 1H), 4.43 (d, *J* = 6.8 Hz, 2H), 4.21 (t, *J* = 6.8 Hz, 1H), 3.71 (t, *J* = 5.1 Hz, 2H), 3.35 (q, *J* = 5.4 Hz, 2H); ^13^C NMR (126 MHz, Chloroform-*d*) δ 143.9, 141.3, 127.7, 127.1, 125.0, 120.0, 66.8, 62.3, 47.3, 43.5.

#### ***N***-Trityl-(***S***)-serine (4)^34^

To a suspension of (S)-Serine (15.00 g, 142.7 mmol) in DCM (300 mL) under a nitrogen atmosphere, TMS-Cl (58 mL, 456.8 mmol) was added dropwise. The mixture was refluxed for 20 min, after which it was cooled to 0 °C. A solution of Et_3_N (66 mL, 473.5 mmol) in DCM (300 mL) was added slowly and the mixture was stirred at room temperature for 45 min. The mixture was cooled to 0 °C and MeOH (8 mL) was added dropwise. The mixture was warmed up to room temperature, after which Et_3_N (20 mL, 143.5 mmol) and Trt-Cl (39.79 g, 142.7 mmol) were added. The mixture was stirred at room temperature for 40 h, after which Et_3_N (100 mL) and MeOH (700 mL) were added. The solvents were removed in vacuo to yield an orange residue. This was dissolved in EtOAc (500 mL) and ice cold 5% aq. citric acid (300 mL). The organic layer was extracted with 2 M aq. NaOH (3 × 150 mL) and washed with water (3 × 150 mL). The combined aqueous phases was washed with EtOAc (150 mL) and neutralized with glacial acetic acid (20 mL). The aqueous phase was extracted with EtOAc (6 × 250 mL), after which the organic layer was washed with brine (400 mL). The organic layer was dried over Na_2_SO_4_, filtered, and the solvent was removed in vacuo to yield a light yellow solid (24.21 g, 49%). Rf 0.26 (1:9 MeOH:DCM); mp 143 °C (dec.) (lit. 110 °C); ^1^H NMR (500 MHz, Chloroform-*d*) δ 7.43–7.38 (m, 6H), 7.26–7.22 (m, 6H), 7.21–7.16 (m, 3H), 3.62 (dd, *J* = 10.8, 3.0 Hz, 1H), 3.43 (dd, *J* = 4.4, 3.0 Hz, 1H), 2.80 (dd, *J* = 11.0, 4.4 Hz, 1H); ^13^C NMR (126 MHz, Chloroform-*d*) δ 175.4, 144.2, 128.6, 127.9, 127.2, 72.3, 62.7, 59.2.

#### ***N***-Trityl-(***S***)-serine allyl ester (5)^34^

To a solution of **4** (18.9 g, 54.4 mmol) in MeOH (190 mL) was added Cs_2_CO_3_ (8.9 g, 27.3 mmol) in portions. The solution was stirred at room temperature for 10 min, after which the solvent was removed *in vacuo* to yield a beige solid. This was dissolved in DMF (38 mL), after which allyl bromide (4.7 mL, 54.3 mmol) was added dropwise. The mixture was stirred at room temperature for 18 h, after which the solvent was removed in vacuo. The residue was dissolved in EtOAc (190 mL) and washed with 5% aq. citric acid (625 mL). The combined aqueous layers were extracted with EtOAc (4 × 300 mL) and the combined organic layers were washed with water (12 × 625 mL). The organic layer was dried over Na_2_SO_4_ and the solvent was removed *in vacuo* to yield yellow oil (18.11 g, 86%). Rf 0.59 (1:4 EtOAc:heptane); ^1^H NMR (500 MHz, Chloroform-*d*) δ 7.52–7.45 (m, 6H), 7.27 (td, *J* = 7.4, 1.4 Hz, 6H), 7.24–7.16 (m, 3H), 5.69 (ddt, *J* = 17.2, 10.4, 5.9 Hz, 1H), 5.23–5.15 (m, 2H), 4.43 (dd, *J* = 10.0, 4.2 Hz, 1H), 4.23 (dd, *J* = 10.1, 6.1 Hz, 1H), 4.22–4.17 (m, 1H), 4.04 (ddt, *J* = 13.0, 5.9, 1.4 Hz, 1H), 3.67 (s, 1H); ^13^C NMR (126 MHz, Chloroform-*d*) δ 171.3, 145.3, 131.4, 128.6, 128.1, 127.9, 118.8, 77.2, 71.2, 70.7, 66.1, 55.8.

#### (*S*)-Allyl 1-tritylaziridine-2-carboxylate (6)

To a solution of **5** (28.00 g, 72.3 mmol) in THF (400 mL) was added Et_3_N (22.2 mL,159.0 mmol) dropwise at 0 °C. MsCl (60.9 mL, 787.7 mmol) was added slowly at 0 °C and the mixture was stirred at 0 °C for 30 min and refluxed for 46 h. The solvent was removed in vacuo and the resulting residue was dissolved in EtOAc (500 mL). The organic layer was washed with 5% aq. citric acid (3 × 250 mL), water (2 × 250 mL), sat. aq. NaHCO_3_ (3 × 250 mL), water (2 × 250 mL) and brine (250 mL). The organic layer was dried over Na_2_SO_4_ and the solvent was removed in vacuo to yield a yellow liquid. The crude product was purified by column chromatography (1:4 EtOAc:heptane) to yield a colourless oil (22.21 g, 83%) Rf 0.56 (1:4 EtOAc:heptane); ^1^H NMR (500 MHz, Chloroform-*d*) δ 7.50 (dd, *J* = 7.3, 1.9 Hz, 6H), 7.30–7.25 (m, 6H), 7.24–7.19 (m, 3H), 5.94 (ddt, *J* = 17.3, 10.4, 5.8 Hz, 1H), 5.42–5.19 (m, 2H), 4.67 (ddt, *J* = 6.0, 3.1, 1.4 Hz, 2H), 2.27 (dd, *J* = 2.7, 1.6 Hz, 1H), 1.91 (dd, *J* = 6.1, 2.7 Hz, 1H), 1.41 (dd, *J* = 6.2, 1.6 Hz, 1H); ^13^C NMR (126 MHz, Chloroform-*d*) δ 171.2, 143.6, 132.0, 129.4, 127.9, 127.7, 127.3, 127.0, 118.6, 74.4, 65.6, 31.8, 28.8.

#### (*S*)-Allyl 1-benzyloxycarbonylaziridine-2-carboxylate (7)

Aziridine **6** (1.397 g, 3.78 mmol) was dissolved in 1:1 MeOH:DCM (12 mL). TFA (6 mL) was added slowly, after which the mixture was stirred for 30 min. The solvent was removed in vacuo and the residue was dissolved in Et_2_O (20 mL) and water (20 mL). The organic layer was extracted with water (3 × 5 mL), the combined aqueous layers were made alkaline at 0 °C with solid NaHCO_3_. EtOAc (35 mL) and benzyl chloroformate (0.54 mL, 3.78 mmol) were added and the mixture was stirred for 16 h. The aqueous layer was washed with EtOAc (3 × 5 mL) and the combined organic layers were washed with 1 M HCl (2 × 20 mL), sat. aq. NaHCO_3_ (2 × 20 mL) and brine (20 mL). The organic layer was dried over Na_2_SO_4_ and the solvent was removed in vacuo. The crude product was purified by column chromatography (1:4 EtOAc:heptane) to yield a colorless oil (318 mg, 32%). Rf 0.36 (1:4 EtOAc:heptane); ^1^H NMR (500 MHz, Chloroform-*d*) δ 7.38–7.33 (m, 5H), 5.88 (ddt, *J* = 17.2, 10.4, 5.8 Hz, 1H), 5.37–5.22 (m, 2H), 5.14 (d, *J* = 7.5 Hz, 2H), 4.61 (tt, *J* = 5.8, 1.4 Hz, 2H), 3.13 (dd, *J* = 5.4, 3.2 Hz, 1H), 2.61 (dd, *J* = 3.1, 1.3 Hz, 1H), 2.49 (dd, *J* = 5.3, 1.4 Hz, 1H); ^13^C NMR (126 MHz, Chloroform-*d*) δ 167.9, 160.7, 135.3, 131.2, 128.58, 128.55, 119.2, 68.6, 66.4, 34.9, 31.4.

#### (*S*)-Allyl 2-(benzyloxycarbonylamino)-3-(9-fluorenylmethoxycarbonylaminoethoxy)propanoate (8)

Aziridine **7** (1.528 g, 5.85 mmol) and 2-(9-fluorenylmethoxycarbonylamino)ethanol (11.27 g, 39.8 mmol) were suspended in toluene (100 mL). BF_3_^**.**^Et_2_O (0.36 mL, 2.92 mmol) was added dropwise, after which the mixture was refluxed for 3 h. The solvent was removed in vacuo and the resulting residue was dissolved in DCM (100 mL). The organic layer was washed with sat. aq. NaHCO_3_ (3 × 50 mL) and brine (50 mL). The organic layer was dried over Na_2_SO_4_ and the solvent was removed in vacuo. The crude product was purified by column chromatography (1:4 EtOAc:heptane) to yield a white solid (548 mg, 17%). Rf 0.47 (1:1 EtOAc:heptane); ^1^H NMR (500 MHz, Chloroform-*d*) δ 7.75 (d, *J* = 7.5 Hz, 2H), 7.58 (d, *J* = 7.5 Hz, 2H), 7.47–7.21 (m, 9H), 5.87 (ddq, *J* = 16.2, 10.9, 6.4, 5.6 Hz, 1H), 5.67 (d, *J* = 8.5 Hz, 1H), 5.41–5.18 (m, 2H), 5.13 (s, 3H), 4.65 (qd, *J* = 13.1, 5.8 Hz, 2H), 4.54 (dd, *J* = 8.2, 4.0 Hz, 1H), 4.51–4.32 (m, 2H), 4.19 (t, *J* = 7.1 Hz, 1H), 3.96–3.85 (m, 1H), 3.73 (dd, *J* = 9.8, 3.2 Hz, 1H), 3.58–3.44 (m, 1H), 3.34 (t, *J* = 5.4 Hz, 2H); ^13^C NMR (126 MHz, Chloroform-*d*) δ 170.1, 156.5, 156.0, 143.9, 141.3, 136.1, 131.4, 128.5, 128.3, 128.1, 127.7, 127.1, 125.1, 112.0, 118.9, 70.9, 70.5, 67.2, 66.7, 66.2, 54.5, 47.3, 40.6. HRMS (ESI+): calcd. for C_31_H_32_N_2_NaO_7_ [M+Na^+^]: 567.2107, found 567.2099.

#### (*S*)-Allyl 2-(benzyloxycarbonylamino)-3-(*tert*-butoxycarbonylaminoethoxy)propanoate (9)

A solution of **8** (367 mg, 0.67 mmol) in 1:4 piperidine:DMF (25 mL) was stirred at room temperature for 30 min. The solvent was removed in vacuo and the residue was dissolved in DCM (50 mL). Boc anhydride (1.31 g, 6.00 mmol) and Et_3_N (0.14 mL, 1.00 mmol) were added and the mixture was stirred at room temperature for 6.5 h. The solvent was removed in vacuo and the residue was dissolved in EtOAc (50 mL). The organic layer was washed with 1 M HCl (3 × 50 mL), sat. aq. NaHCO_3_ (2 × 50 mL) and brine (50 mL). The organic layer was dried over Na_2_SO_4_ and the solvent was removed in vacuo. The crude product was purified by column chromatography (1:4 EtOAc:heptane) to yield a colorless oil (140 mg, 49%). Rf 0.53 (1:9 MeOH:DCM); ^1^H NMR (500 MHz, Chloroform-*d*) δ 7.41–7.30 (m, 5H), 5.91 (ddt, *J* = 16.2, 10.7, 5.6 Hz, 1H), 5.68 (d, *J* = 8.7 Hz, 1H), 5.45–5.23 (m, 2H), 5.13 (s, 2H), 4.81 (s, 1H), 4.67 (qd, *J* = 13.2, 5.8 Hz, 2H), 4.53 (dt, *J* = 8.7, 3.3 Hz, 1H), 3.89 (dd, *J* = 9.5, 3.4 Hz, 1H), 3.72 (dd, *J* = 9.5, 3.2 Hz, 1H), 3.56–3.41 (m, 2H), 3.26 (q, *J* = 5.5 Hz, 2H), 1.43 (s, 9H); ^13^C NMR (126 MHz, Chloroform-*d*) δ 167.0, 156.0, 155.9, 136.2, 131.5, 128.6, 128.3, 128.2, 119.0, 79.4, 70.8, 70.7, 67.1, 66.2, 54.5, 40.1, 28.4. HRMS (ESI+): calcd. for C_21_H_30_N_2_NaO_7_ [M+Na^+^]: 445.1951, found 445.1967.

#### (*S*)-2-(((9*H*-Fluoren-9-yl)methoxy)carbonylamino)-3-(*tert*-butoxycarbonylaminoethoxy)propanoate (1)

To a solution of **9** (310 mg, 0.73 mmol) in MeOH (50 mL) was added ammonium formate (463 mg, 7.34 mmol) and the solution was purged with N_2_ for 10 min. Pd/C (273 mg, 2.57 mmol, 10% loading by weight on wet support) was added and the mixture was stirred for 16 h. The mixture was filtered through a pad of celite and the filtrate was concentrated in vacuo. The resulting residue was dissolved in 2:1 acetone:sat. aq. NaHCO_3_ (25 mL) and Fmoc-OSu (240 mg, 0.71 mmol) was added. After stirring for 5 h the acetone was removed in vacuo and EtOAc (25 mL) was added. The solution was acidified with 0.5 M KHSO4, the layers were separated and the aqueous layer was extracted with EtOAc (3 × 25 mL). The combined organic layers were dried over MgSO_4_ and concentrated in vacuo to yield a white solid (260 mg, 75%). Rf 0.45 (95:5:1 DCM:MeOH:AcOH); ^1^H NMR (500 MHz, Methanol-d4) δ 7.80 (d, *J* = 7.5 Hz, 2H), 7.72–7.66 (m, 2H), 7.39 (td, *J* = 7.5, 0.9 Hz, 2H), 7.32 (td, *J* = 7.5, 1.2 Hz, 2H), 4.47–4.31 (m, 3H), 4.25 (s, 1H), 3.84 (d, *J* = 5.0 Hz, 1H), 3.74 (d, *J* = 3.6 Hz, 1H), 3.49 (d, *J* = 5.7 Hz, 2H), 3.28–3.12 (m, 2H), 1.42 (s, 9H); ^13^C NMR (126 MHz, Methanol-d4) δ 173.8, 158.7, 145.4, 145.2, 142.6, 128.8, 128.19, 128.17, 126.31, 126.25, 120.9, 71.7, 71.5, 55.9, 48.4, 41.2, 28.8. HRMS (ESI+): calcd. for C_25_H_30_N_2_NaO_7_ [M+Na^+^]: 493.1951, found 493.1940.

#### Phthalimidoacetaldehyde^40^

A solution of 2-(pthalimido) acetaldehydediethylacetal (7.0 g, 26.6 mmol) in 2:1 CHCl_3_:TFA (150 mL) was stirred for 1 h at 0 °C followed by stirring for 5 h at rt. The solvent was removed in vacuo and the remaining traces of TFA were co-evaporated with DCM to yield an white solid (5.0 g, 100%). Rf 0.20 (1:1 EtOAc:heptane); ^1^H NMR (500 MHz, Chloroform-*d*) δ 9.65 (s, 1H), 7.89 (dd, *J* = 5.5, 3.1 Hz, 2H), 7.76 (dd, *J* = 5.5, 3.0 Hz, 2H), 4.56 (s, 2H); ^13^C NMR (126 MHz, Chloroform-*d*) δ 193.7, 167.7, 134.5, 132.0, 123.8, 47.5.

#### Methyl (***S***)-2-(((benzyloxy)carbonyl)amino)-3-(chloro-l5-azaneyl)propanoate (11)^35^

(S)-3-Amino-2-(((benzyloxy)carbonyl)amino)propanoic acid (3.0 g, 12.6 mmol) was dissolved in MeOH (20 mL) and 4 M HCl in dioxane (15.7 mL, 63.0 mmol) was added. The resulting solution was stirred for 16 h and concentrated *in vacuo* to obtain a white solid (3.64 g, 100%). ^1^H NMR (400 MHz, DMSO-*d6*) δ 8.67–8.25 (m, 3H), 7.96 (d, *J* = 8.2 Hz, 1H), 7.44–7.27 (m, 5H), 5.06 (s, 2H), 4.47 (td, *J* = 8.8, 4.7 Hz, 1H), 3.67 (s, 3H), 3.21 (dd, *J* = 13.0, 4.8 Hz, 1H), 3.07 (dd, *J* = 13.1, 9.2 Hz, 1H); ^13^C NMR (101 MHz, DMSO-*d6*) δ 169.8, 156.1, 136.6, 128.4, 127.9, 127.8, 65.9, 52.6, 51.8. MS (ESI+): calcd for C_12_H_17_N_2_NaO_4_ (M+Na^+^): 276.11, found 276.13.

#### (*S*)-2-(((Benzyloxy)carbonyl)amino)-3-((tert-butoxycarbonyl)(2-(1,3-dioxoisoindolin-2-yl)ethyl)amino)propanoic acid (12)

To a solution of amine of **11** (2.12 g, 8.4 mmol) in MeOH (20 mL) was added phthalimidoacetaldehyde (1.90 g, 10.1 mmol) and the mixture was allowed to react for 30 min. NaBH_3_CN (1.05 g, 16.8 mmol) was added portion wise and the mixture was stirred for 16 h. After completion the solvent was removed in vacuo and the resulting residue was dissolved in THF (20 mL), Boc anhydride (2.75 g, 12.6 mmol) was added followed by the addition of Et_3_N (1.4 mL, 10.1 mmol). After stirring for 4 h the solvent was removed in vacuo. The residue was dissolved in EtOAC (20 mL), washed with 0.1 M KHSO_4_ (2 × 10 mL) and brine (10 mL), dried over MgSO_4_ and concentrated in vacuo. The crude product was purified by column chromatography (1:4 EtOAc:heptane) to yield a white solid (3.5 g, 81%). Rf 0.42 (1:1 EtOAc:Heptane); ^1^H NMR (500 MHz, DMSO-*d*_6_, mixture of rotamers) δ 7.93–7.67 (m, 5H), 7.42–7.23 (m, 5H), 5.10–4.96 (m, 2H), 4.50–4.23 (m, 1H), 3.72 (ddd, *J* = 14.1, 8.0, 4.2 Hz, 1H), 3.67–3.59 (m, 4H), 3.59–3.49 (m, 1H), 3.32–3.22 (m, 1H), 1.18–0.93 (m, 9H); ^13^C NMR (126 MHz, DMSO-*d6*, mixture of rotamers) δ 156.5, 155.1, 154.9, 137.4, 137.3, 135.0, 134.7, 132.2, 132.1, 128.8, 128.3, 128.1, 123.6, 123.4, 135.0, 79.6, 79.5, 66.1, 53.5, 52.7, 52.5, 51.8, 47.4, 46.9, 45.6, 45.0, 35.8, 28.0, 27.8; HRMS (ESI+): calcd. for C_27_H_31_N_3_NaO_6_ [M+Na^+^]: 548.2009, found 548.2019.

#### (*S*)-2-(((Benzyloxy)carbonyl)amino)-3-((tert-butoxycarbonyl)(2-((tert-butoxycarbonyl)amino)ethyl)amino)propanoic acid (13)

To a solution of **12** (1.50 g, 2.85 mmol) in water:MeOH (10 ml) was added NaOH (120 mg, 3.00 mmol) and the resulting mixture was stirred for 5 h. The solvent was removed *in vacuo* and the residue was dissolved in EtOAc (30 mL). The organic phase was washed with 0.1 M KHSO_4_ (2 × 10 mL), dried over MgSO_4_ and concentrated *in vacuo* to obtain the free acid. Without further purification the residue was dissolved in EtOH (30 mL) and N_2_H_4_^**.**^H_2_O (0.58 mL, 7.80 mmol) was added. The resulting solution heated to 70 °C and stirred for 3 h. After cooling to rt the insoluble content was removed by filtration, and washed with DCM. The filtrate was concentrated in vacuo to yield the primary amine as an off white solid. The crude amine was dissolved in 1:1 THF:water (20 mL), NaHCO_3_ (530 mg, 6.3 mmol) and Boc anhydride (1.24 g, 5.7 mmol). After stirring for 6 h the solvent was removed *in vacuo*. The residue was dissolved in EtOAC (20 mL), washed with 0.1 M KHSO_4_ (2 × 10 mL) and brine (10 mL), dried over MgSO_4_ and concentrated *in vacuo*. The resulting residue was purified by column chromatography (98:2:1 DCM:MeOH:AcOH) to yield a white solid (737 mg, 54% over three steps). Rf 0.52 (95:5:1 DCM:MeOH:AcOH); ^1^H NMR (500 MHz, Methanol-*d4*) δ 7.42–7.21 (m, 5H), 5.10 (dq, *J* = 21.9, 12.1, 10.0 Hz, 2H), 4.52 (dd, *J* = 9.3, 5.4 Hz, 1H), 3.87–3.65 (m, 1H), 3.60–3.35 (m, 2H), 3.27–3.19 (m, 1H), 3.16 (t, *J* = 6.2 Hz, 2H), 1.50–1.22 (m, 18H); ^13^C NMR (126 MHz, Methanol-*d4*) δ 173.7, 158.4, 157.7, 157.2, 138.1, 129.5, 129.0, 128.9, 81.84, 81.84, 80.1, 67.7, 54.5, 54.3, 50.5, 49.5, 39.9, 39.5, 28.8, 28.6; HRMS (ESI+): calcd. for C_23_H_35_N_3_NaO_8_ [M+Na^+^]: 504.2322, found 504.2345.

#### (*S*)-2-((((9H-Fluoren-9-yl)methoxy)carbonyl)amino)-3-((tert-butoxycarbonyl)(2-((tert-butoxycarbonyl)amino)ethyl)amino)propanoic acid (2)

To a solution of **13** (300 mg, 0.62 mmol) in MeOH (20 mL) was purged with N_2_ for 10 min. Pd/C (33 mg, 0.03 mmol, 10% loading by weight) was added, the atmosphere was exchanged for H_2_ and the mixture was stirred for 16 h. The mixture was filtered through a pad of celite and the filtrate was concentrated in vacuo. The resulting residue was dissolved in 2:1 acetone:sat. aq. NaHCO_3_ (25 mL) and Fmoc-OSu (300 mg, 0.61 mmol) was added. After stirring for 5 h the acetone was removed *in vacuo* and EtOAc (25 mL) was added. The solution was acidified with 0.5 M KHSO_4_, the layers were separated and the aqueous layer was extracted with EtOAc (3 × 25 mL). The combined organic layers were dried over MgSO_4_ and concentrated *in vacuo* to yield a white solid (260 mg, 73%); Rf 0.31 (80:20:1 EtOAc:Heptane:AcOH). ^1^H NMR (500 MHz, Methanol-*d*_4_) δ 7.79 (d, *J* = 7.5 Hz, 2H), 7.66 (dd, *J* = 7.6, 3.9 Hz, 2H), 7.39 (t, *J* = 7.4 Hz, 2H), 7.31 (td, *J* = 7.6, 1.3 Hz, 2H), 4.52 (dd, *J* = 9.1, 5.2 Hz, 1H), 4.35 (dt, *J* = 8.2, 4.1 Hz, 2H), 4.22 (t, *J* = 6.9 Hz, 1H), 3.77 (dd, *J* = 26.4, 14.5 Hz, 1H), 3.55–3.33 (m, 2H), 3.29–3.07 (m, 3H), 1.46 (s, 9H), 1.43 (s, 9H). ^13^C NMR (126 MHz, Methanol-*d*_4_) δ 173.7, 158.5, 145.3, 145.2, 142.6, 128.79, 128.77, 126.2, 120.9, 81.9, 81.7, 68.1, 54.6, 50.5, 49.8, 48.8, 39.9, 39.5, 28.8, 28.7. HRMS (ESI+): calcd for C_30_H_39_N_3_NaO_8_ [M+Na^+^]: 592.2635, found 592.2661.

#### Synthesis of histone peptides

All histone peptides were manually synthesized using standard solid-phase method as previously described^17–19,21,22^. The peptide sequences H4K20 (13–27 aa) and H3K9 (1–15 aa) were assembled on a Wang resin (0.21 mmol scale), and the peptide chain (1–15 aa) H3K4 was assembled on Breipohl resin (0.21 mmol scale) with Fmoc/^t^Bu strategy. Side chain protection was as follows: Arg(Pbf), Thr(tBu), Gln(Trt), Lys(Boc), azalysine(Boc)_2_, oxolysine(Boc), Ser(tBu). Coupling of each amino acid was performed in the presence of 3 mol excess of Fmoc-amino acid, 3.6 mol excess of 1 M HOBt and 3.3 mol excess of 1 M DIPCDI. Coupling of the specific unnatural amino acids was achieved in the presence of 1.5 mol excess, and 7.2 mol excess of 1 M HOBt and 6.6 mol excess of 1 M DIPCDI for 16 h. The successful coupling reactions and Fmoc deprotections were qualitatively confirmed by the colour Kaiser test^41,42^. After the final Fmoc deprotection, the cleavage step from the resin of all remained protecting groups was done in a standard cocktail containing TFA/TIPS/H_2_O (95:2.5:2.5).

Histones H3 and H4 crude peptides were purified by prep-HPLC on a Phenomenex Gemini-NX 3u C18 110 Å reversed-phase column (150 × 21.2 mm). Solvent A is 0.1% trifluoroacetic acid in H_2_O, Solvent B is 0.1% trifluoroacetic acid in acetonitrile. The pure fractions containing product were combined, frozen, and freeze-dried overnight to produce highly pure histone peptides as a white-off solid. The success of the synthesis of histone peptides was analyzed by analytical HPLC to confirm the high purity and LC–MS and MALDI-TOF MS to confirm the identity. Results of characterization of substrate peptides are clarified in the Supplementary Information (Supplementary Figs. S4–S6).

## Supplementary Information

Supplementary Information.

## References

[1] Bannister AJ, Kouzarides T (2011). Regulation of chromatin by histone modifications. Cell Res..

[2] Cornett EM (2018). A functional proteomics platform to reveal the sequence determinants of lysine methyltransferase substrate selectivity. Sci. Adv..

[3] Kouzarides T (2007). Chromatin modifications and their function. Cell.

[4] Strahl BD, Allis CD (2000). The language of covalent histone modifications. Nature.

[5] Black JC, Van Rechem C, Whetstine JR (2012). Histone lysine methylation dynamics: Establishment, regulation, and biological impact. Mol. Cell.

[6] Shahbazian MD, Grunstein M (2007). Functions of site-specific histone acetylation and deacetylation. Annu. Rev. Biochem..

[7] Luo M (2018). Chemical and biochemical perspectives of protein lysine methylation. Chem. Rev..

[8] Martin C, Zhang Y (2005). The diverse functions of histone lysine methylation. Nat. Rev. Mol. Cell Biol..

[9] Kaniskan HÜ, Konze KD, Jin J (2015). Selective inhibitors of protein methyltransferases. J. Med. Chem..

[10] Copeland RA (2018). Protein methyltransferase inhibitors as precision cancer therapeutics: A decade of discovery. Philos. Trans. R. Soc. Lond. B Biol. Sci..

[11] Kaniskan H, Martini ML, Jin J (2018). Inhibitors of protein methyltransferases and demethylases. Chem. Rev..

[12] Guo H-B, Guo H (2007). Mechanism of histone methylation catalyzed by protein lysine methyltransferase SET7/9 and origin of product specificity. Proc. Natl. Acad. Sci. USA.

[13] Schapira M (2011). Structural chemistry of human SET domain protein methyltransferases. Curr. Chem. Genom..

[14] Linscott JA (2016). Kinetic isotope effects reveal early transition state of protein lysine methyltransferase SET8. Proc. Natl. Acad. Sci. USA.

[15] Poulin MB (2016). Transition state for the NSD2-catalyzed methylation of histone H3 lysine 36. Proc. Natl. Acad. Sci. USA.

[16] Couture J-F, Dirk LMA, Brunzelle JS, Houtz RL, Trievel RC (2008). Structural origins for the product specificity of SET domain protein methyltransferases. Proc. Natl. Acad. Sci. USA.

[17] Al Temimi AHK (2019). Importance of the main chain of lysine for histone lysine methyltransferase catalysis. Org. Biomol. Chem..

[18] Al Temimi AHK (2019). γ-Thialysine versus lysine: An insight into the epigenetic methylation of histones. Bioconjugate Chem..

[19] Belle R (2017). Investigating d-lysine stereochemistry for epigenetic methylation, demethylation and recognition. Chem. Commun..

[20] Temimi AHKA (2020). Examining sterically demanding lysine analogs for histone lysine methyltransferase catalysis. Sci. Rep.

[21] Al Temimi AHK (2019). The nucleophilic amino group of lysine is central for histone lysine methyltransferase catalysis. Commun. Chem..

[22] Temimi AHKA (2017). Lysine possesses the optimal chain length for histone lysine methyltransferase catalysis. Sci. Rep..

[23] Al Temimi AHK (2020). Methylation of geometrically constrained lysine analogues by histone lysine methyltransferases. Chem. Commun..

[24] Al Temimi AHK (2020). Lysine ethylation by histone lysine methyltransferases. ChemBioChem.

[25] Blum G, Bothwell IR, Islam K, Luo M (2013). Profiling protein methylation with cofactor analog containing terminal alkyne functionality. Curr. Protoc. Chem. Biol..

[26] Bothwell IR (2012). Se-Adenosyl-l-selenomethionine cofactor analogue as a reporter of protein methylation. J. Am. Chem. Soc..

[27] Bothwell IR, Luo M (2014). Large-scale, protection-free synthesis of Se-adenosyl-l-selenomethionine analogues and their application as cofactor surrogates of methyltransferases. Org. Lett..

[28] Islam K, Zheng W, Yu H, Deng H, Luo M (2011). Expanding cofactor repertoire of protein lysine methyltransferase for substrate labeling. ACS Chem. Biol..

[29] Peters W (2010). Enzymatic site-specific functionalization of protein methyltransferase substrates with alkynes for click labeling. Angew. Chem. Int. Ed..

[30] Wang R (2013). Profiling genome-wide chromatin methylation with engineered posttranslation apparatus within living cells. J. Am. Chem. Soc..

[31] Willnow S, Martin M, Lüscher B, Weinhold E (2012). A selenium-based click AdoMet analogue for versatile substrate labeling with wild-type protein methyltransferases. ChemBioChem.

[32] Liu H, Pattabiraman VR, Vederas JC (2007). Stereoselective syntheses of 4-Oxa diaminopimelic acid and its protected derivatives via aziridine ring opening. Org. Lett..

[33] Maynard SJ, Almeida AM, Yoshimi Y, Gellman SH (2014). New charge-bearing amino acid residues that promote β-sheet secondary structure. J. Am. Chem. Soc..

[34] Kelleher F, Proinsias KÓ (2007). Use of the Mitsunobu reaction in the synthesis of orthogonally protected α, β-diaminopropionic acids. Tetrahedron Lett..

[35] Chhabra SR, Mahajan A, Chan WC (2002). Homochiral 4-azalysine building blocks: Syntheses and applications in solid-phase chemistry. J. Org. Chem..

[36] Shinkai Y, Tachibana M (2011). H3K9 methyltransferase G9a and the related molecule GLP. Genes Dev..

[37] Wu H (2010). Structural biology of human H3K9 methyltransferases. PLoS One.

[38] Xiao B (2003). Structure and catalytic mechanism of the human histone methyltransferase SET7/9. Nature.

[39] Liu W, Chan ASH, Liu H, Cochrane SA, Vederas JC (2011). Solid supported chemical syntheses of both components of the lantibiotic lacticin. J. Am. Chem. Soc..

[40] Veale EB, O'Brien JE, McCabe T, Gunnlaugsson T (2008). The synthesis, N-alkylation and epimerisation study of a phthaloyl derived thiazolidine. Tetrahedron.

[41] Kaiser E, Colescott RL, Bossinger CD, Cook PI (1970). Color test for detection of free terminal amino groups in the solid-phase synthesis of peptides. Anal. Biochem..

[42] Sarin VK, Kent SBH, Tam JP, Merrifield RB (1981). Quantitative monitoring of solid-phase peptide synthesis by the ninhydrin reaction. Anal. Biochem..

