# Supplementary material for: Fine-tuning of lysine side chain modulates the activity of histone lysine methyltransferases
Source: Sci Rep. 2020 Dec 9;10:21574. doi: 10.1038/s41598-020-78331-0 (PMC7726145; doi:10.1038/s41598-020-78331-0)
Supplement: Supplementary file 1 — Supplementary Information. [file 41598_2020_78331_MOESM1_ESM.docx]

**Supplementary Information**

**Fine-tuning of lysine side chain modulates the activity of histone lysine methyltransferases**

Abbas H. K. Al Temimi,^1⊥^ Jona Merx, ^1⊥^ Christian J. van Noortwijk,^1^ Giordano Proietti,^2^ Romano Buijs,^1^ Paul B. White,^1^ Floris P. J. T. Rutjes,^1^ Thomas J. Boltje^1^ & Jasmin Mecinović^1,2^*

^1^ Radboud University, Institute for Molecules and Materials, Heyendaalseweg 135, 6525 AJ Nijmegen, The Netherlands

^2^ University of Southern Denmark, Department of Physics, Chemistry and Pharmacy, Campusvej 55, 5230 Odense, Denmark

^⊥^ The authors contributed equally to this work.

* e-mail: mecinovic@sdu.dk

**Table of Contents**

| 1. | General experimental section | 3 |
| --- | --- | --- |
| 2. | Synthetic schemes of histone peptides | 5 |
| 3. | Characterization of histone peptides | 8 |
| 4. | Time course supplementary figures | 11 |
| 5. | MALDI-TOF MS supplementary figures | 16 |
| 6. | Enzyme kinetics supplementary figures | 20 |
| 7. | Inhibition supplementary figures | 21 |
| 8. | NMR supplementary figures | 24 |
| 9. | NMR spectra | 26 |

**1. General experimental section**

**1.1 Methods**

^1^H and ^13^C NMR spectra for the two building blocks and the intermediates were recorded on a Bruker AVANCE III (500 MHz ^1^H, 125 MHz ^13^C) equipped with a Bruker Prodigy BB cryoprobe in the solvent indicated at room temperature. Chemical shifts are reported in *δ* (ppm) units relative to the internal reference tetramethylsilane (Me_4_Si). For ^1^H NMR spectra, the following abbreviations are used to describe multiplicities: s (singlet), d (doublet), t (triplet), bs (broad singlet), dd (double doublet), and m (multiplet). Coupling constants are reported in Hertz (Hz) as a *J* value. Structurally significant resonances for the final three building blocks were assigned by HSQC (^1^H–^13^C) and COSY (^1^H–^1^H) correlations. NMR was carried out in 5 mm diameter Boroeco-5-7 tubes from Deutero (Kastellaun, Germany). NMR spectra were recorded at 298 K unless otherwise specified. Mass spectra were recorded on Thermo Finnigan LCQ Advantage Max. LC-MS was carried out on a Shimadzu LCMS-QP8000 (Duisburg, Germany) single quadrupole bench-top mass spectrometer operating in a positive ionization mode. The scanning range was m/z 50-2000. A gradient of MeCN/H_2_O containing 0.1 % formic acid was used. Samples were injected using a flow rate of 0.2 mL/min and eluted with 5-100 % in 50 min, infused in the Electrospray system. All compounds were routinely checked by TLC on Kieselgel 60 F254 (Merck, Darmstadt, Germany); spots were visualized under UV light (254 nm) and were stained with ninhydrin, 2-4-ninitrophenylhydrazine (DNP), Cerium Molybdate Stain or aqueous KMnO_4_ (depending on the reaction), followed by heating on a hot plate. Rf values were obtained with the indicated solvent mixtures. All solvents were reagent grade and, when necessary, were purified and dried by standard methods. Organic solutions were dried over anhydrous sodium sulfate and anhydrous magnesium sulfate. The yields of the samples were calculated after drying the samples under high vacuum overnight.

**1.2 Materials**

All commercially purchased reagents were used without further purification as delivered from the corresponding companies. Amino acids and the reagents were purchased from the following companies: Preloaded Wang resin (100-200 mesh) as the solid support and Fmoc-Lys(Boc)-OH were obtained from Novabiochem (Darmstad, Germany). Trifluoroacetic acid (TFA), Triisopropylsilane (TIS), 1-Hydroxybenzotriazole (HOBt), Tris-*d*_11_ solution, *S*-Adenosyl-L-Methionine (SAM), *α*-cyano-4-hydroxycinnamic acid, and *N,N′*-diisopropylethylamine (DIPEA), were purchased from Sigma Aldrich. Fmoc-allylglycine-OH, Fmoc-Thr(^t^Bu)-OH, and Fmoc-Gln(Trt)-OH were purchased from Carbosynth (Berkshire, UK). *N*,*N'*-Diisopropylcarbodiimide (DIPCDI) and piperidine were purchased from Biosolve chemicals (Valkenswaard, The Netherlands). Breipohl Resin [Fmoc-4-methoxy-4’-(-carboxypropyloxy)-benzhydrylamine linked to Alanyl-aminomethyl] (200-400 mesh) were purchased from Bachem AG (Bubendorf, Switzerland). Fmoc-Ala.OH.H_2_O and Fmoc-Asn(Trt)-OH were obtained from Iris Biotech (Marktredwitz, Germany). Fmoc-OSu was purchased from Chemicals Block (Santiago, USA). Fmoc-Ser(^t^Bu)-OH, Fmoc-Gly-OH, Fmoc-His(Trt)-OH, were purchased from Chem-Impex Int’l Inc (Illinois, USA). Fmoc-Arg(Pbf)-OH, and 1-[Bis(dimethylamino)methylene]-1H-1,2,3-triazolo[4,5-b]pyridinium 3-oxid hexafluorophosphate (HATU) were obtained from Fluorochem Ltd. (Derbyshire, UK). DMF and MeCN were purchased from Actu-All Chemicals b.v (Oss, The Netherlands).

**2. Synthetic schemes of histone peptides**

**Supplementary Scheme 1.** Solid phase synthesis of the natural histone peptide H3K9. The same synthetic route was used to synthesize unnatural histone peptides that possess varieties at γ-site at position 9 of histone 3.

****

**Supplementary Figure 1.** Histone peptides containing unnatural lysine analogues H3K_O_9 and H3K_N_9.

**Supplementary Scheme 2.** Solid phase synthesis of the natural histone peptide H4K20. The same synthetic route was used to synthesize unnatural histone peptides that possess varieties at γ-site at position 20 of histone 4.

**Supplementary Figure** **2.** Histone peptides containing unnatural lysine analogues H4K_O_20 and H4K_N_20.

**Supplementary Scheme 3.** Solid phase synthesis of the natural histone peptide H3K4. The same synthetic route was used to synthesize unnatural histone peptides that possess varieties at γ-site at position 4 of histone 3.

****

**Supplementary Figure 3.** Histone peptides containing unnatural lysine analogues H3K_O_4 and H3K_N_4.

**3. Characterization of histone peptides**


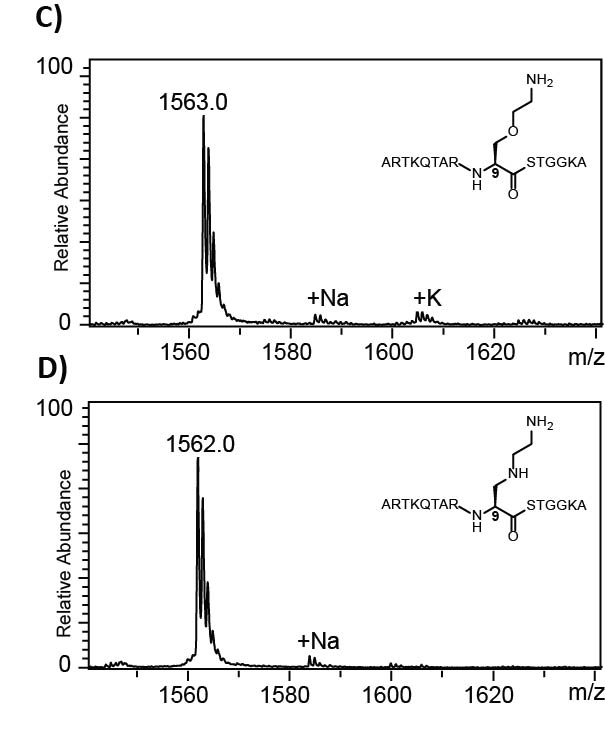


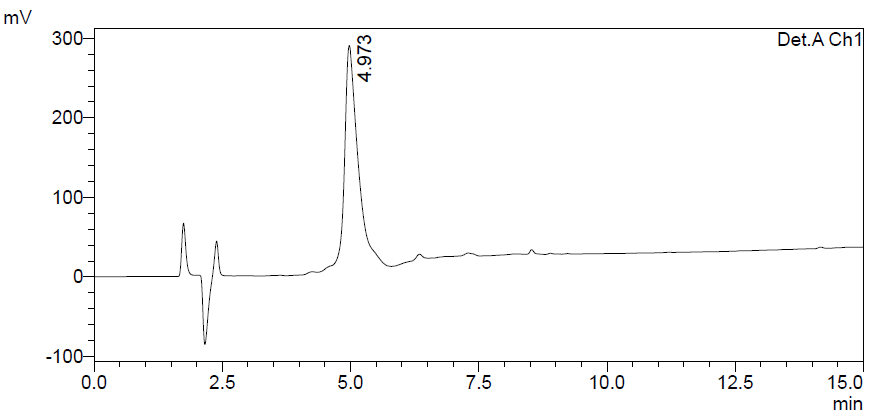

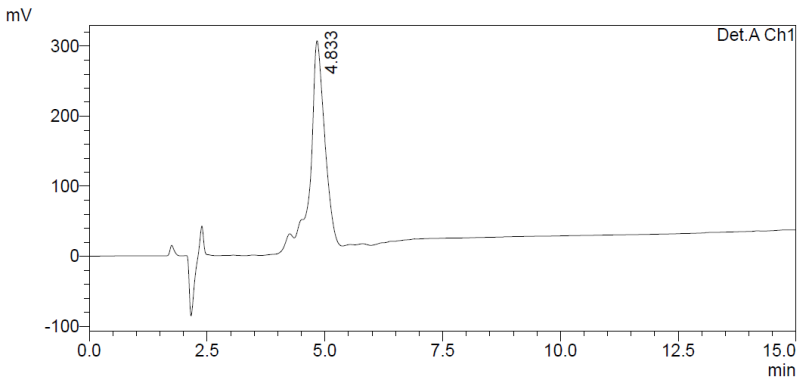


**A)**

**B)**

**Supplementary Figure 4.** Characterization of histone peptides at position 9 after prep-HPLC purification. Analytical HPLC trace of **A**) H3K_O_9 and **B**) H3K_N_9. MALDI-TOF MS of **C**) H3K_O_9 and D) H3K_N_9.


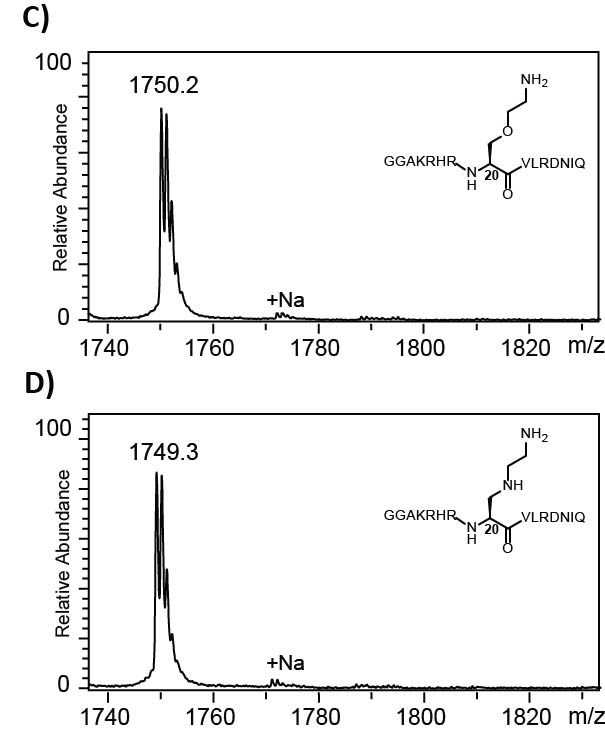

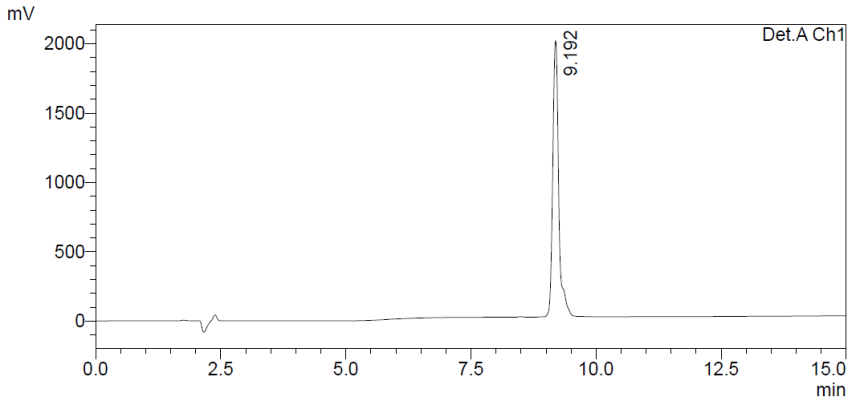


**A)**


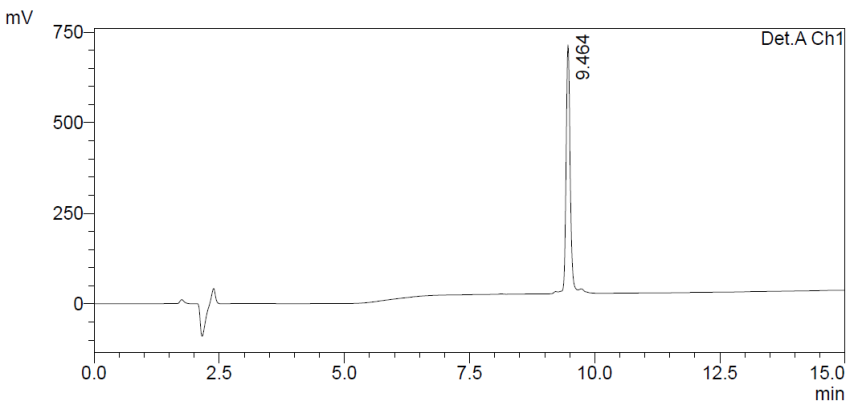


**B)**

**Supplementary Figure 5.** Characterization of histone peptides at position 20 after prep-HPLC purification. Analytical HPLC trace of **A**) H4K_O_20 and B) H4K_N_20. MALDI-TOF MS of **C**) H4K_O_20 and **D**) H4K_N_20.


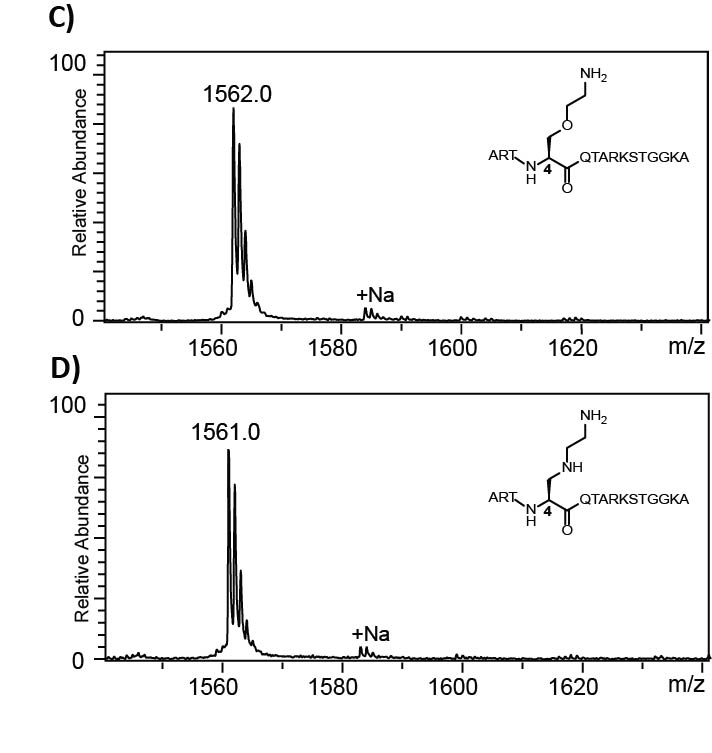

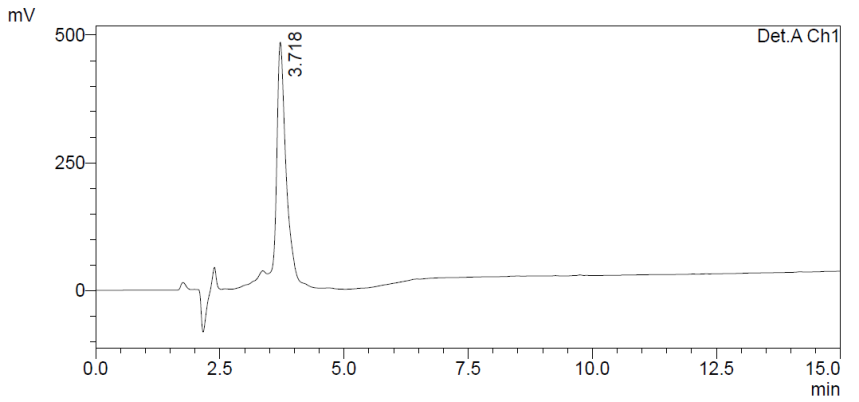

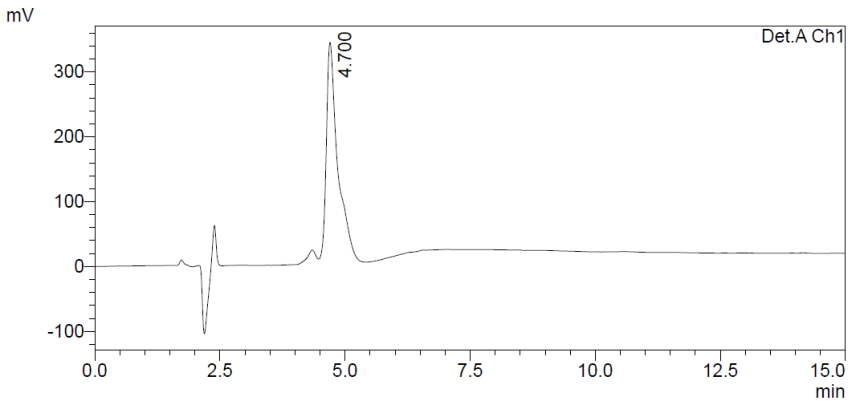


**A)**

**B)**

**Supplementary Figure 6.** Characterization of histone peptides at position 4 after prep-HPLC purification. Analytical HPLC trace of **A**) H3K_O_4 and **B**) H3K_N_4. MALDI-TOF MS of **C**) H3K_O_4 and **D**) H3K_N_4.

**4. Time course supplementary figures**


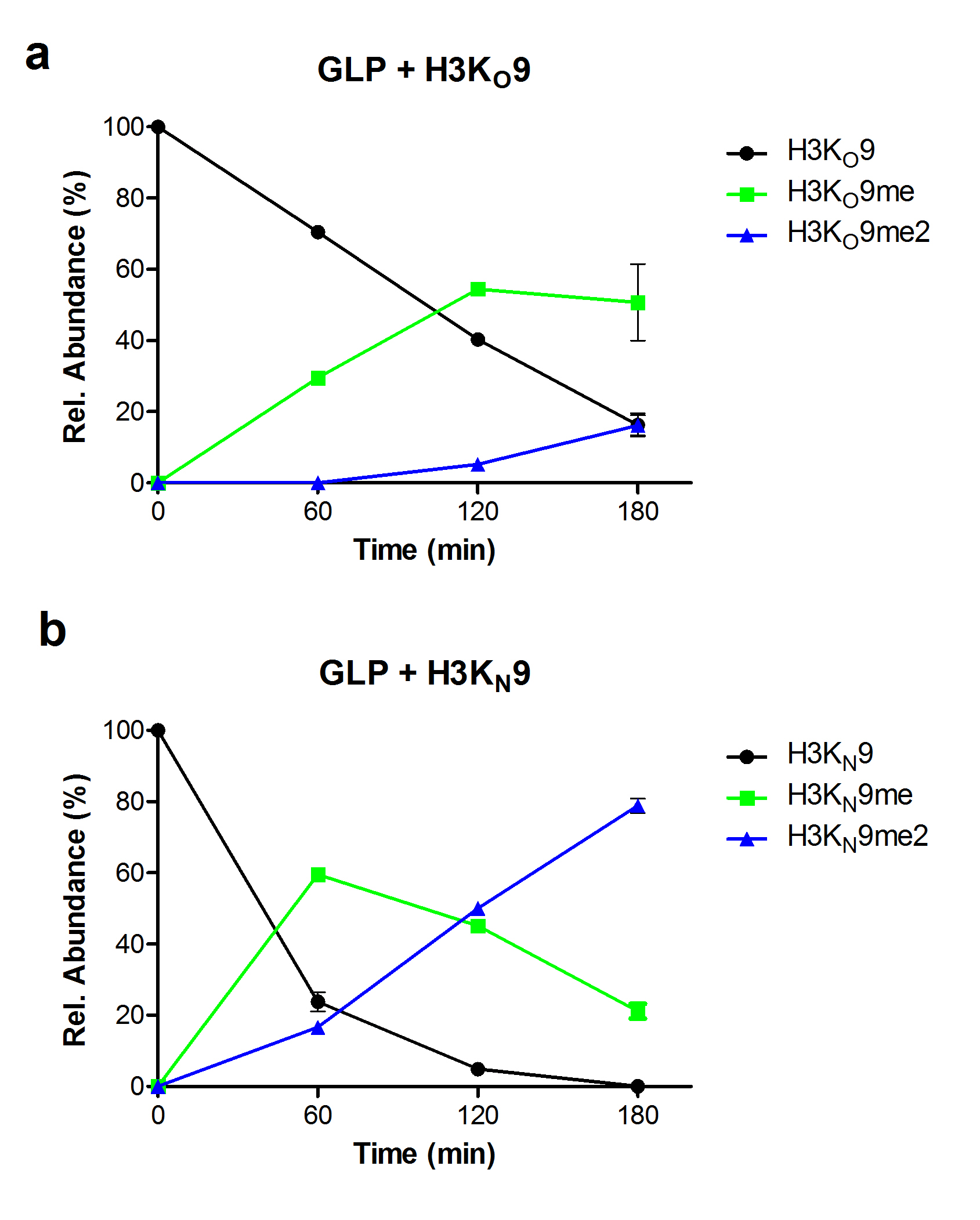


**Supplementary Figure 7.** Time course assays of GLP (2 µM) with **a**) H3K_O_9 and **b**) H3K_N_9 histone peptide substrates. The reactions implemented at 100 µM substrate concentration in the presence of SAM (500 µM) in Tris buffer (pH 8.0) at 37 °C as determined by MALDI-TOF MS. Unmodified (displayed as circles in black line), mono- (displayed as squares in green line) and di- (displayed as blue up-triangle in blue line) represent the unmethylation and methylation states of the substrates and the products based on the mass peak integral at each time point in hours (shown on the x-axis, 0, 1, 2, and 3).


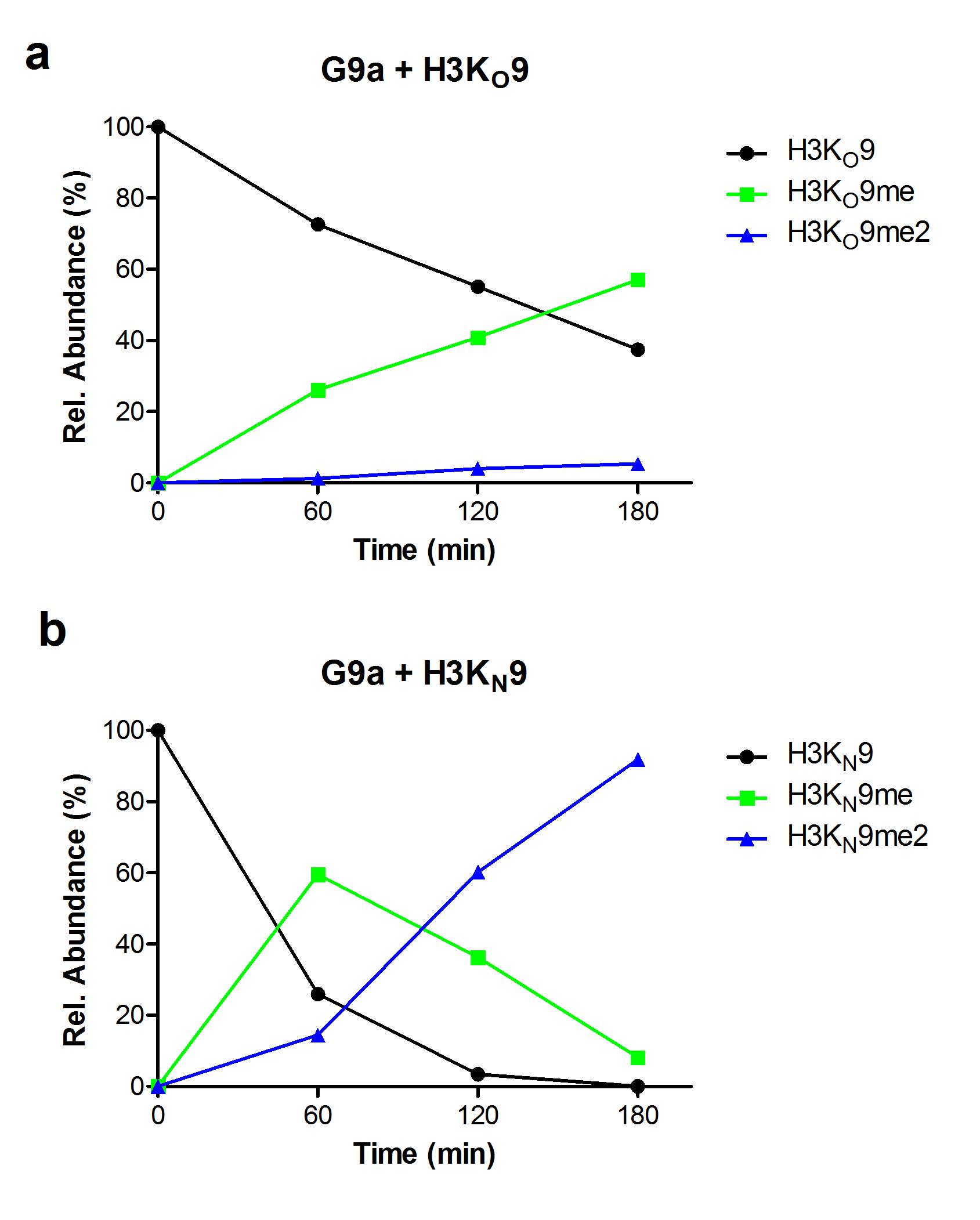


**Supplementary Figure 8.** Time course assays of G9a (2 µM) with **a**) H3K_O_9 and **b**) H3K_N_9 histone peptide substrates. The reactions implemented at 100 µM substrate concentration in the presence of SAM (500 µM) in Tris buffer (pH 8.0) at 37 °C as determined by MALDI-TOF MS. Unmodified (displayed as circles in black line), mono- (displayed as squares in green line) and di- (displayed as blue up-triangle in blue line) represent the unmethylation and methylation states of the substrates and the products based on the mass peak integral at each time point in hours (shown on the x-axis, 0, 1, 2, and 3).


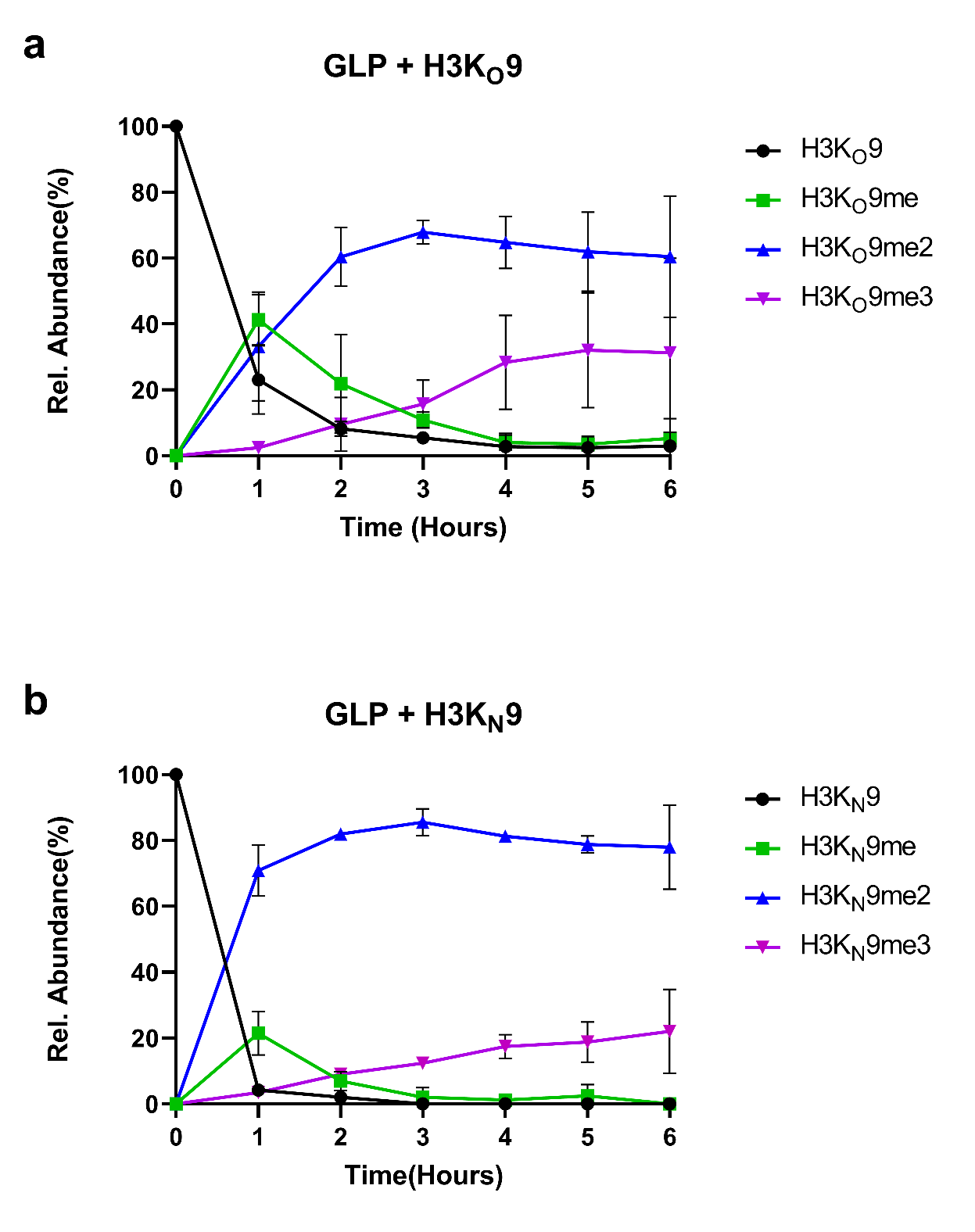


**Supplementary Figure 9.** Time course assays of GLP (10 µM) with **a**) H3K_O_9 and **b**) H3K_N_9 histone peptide substrates. The reactions implemented at 100 µM substrate concentration in the presence of SAM (500 µM) in Tris buffer (pH 8.0) at 37 °C as determined by MALDI-TOF MS. Unmodified (displayed as circles in black line), mono- (displayed as squares in green line), di- (displayed as blue up-triangle in blue line) and tri- (displayed as pink down-triangle in blue line) represent the unmethylation and methylation states of the substrates and the products based on the mass peak integral at each time point in hours (shown on the x-axis, 0, 1, 2, 3, 4, 5 & 6).

**Supplementary Figure 10.** Time course assays of G9a (10 µM) with **a**) H3K_O_9 and **b**) H3K_N_9 histone peptide substrates. The reactions implemented at 100 µM substrate concentration in the presence of SAM (500 µM) in Tris buffer (pH 8.0) at 37 °C as determined by MALDI-TOF MS. Unmodified (displayed as circles in black line), mono- (displayed as squares in green line), di- (displayed as blue up-triangle in blue line) and tri- (displayed as pink down-triangle in pink line) represent the unmethylation and methylation states of the substrates and the products based on the mass peak integral at each time point in hours (shown on the x-axis, 0, 1, 2, 3, 4, 5 & 6).

****

**Supplementary Figure 11.** Stability assay of a) GLP (10 µM) and b) G9a (10 uM) incubated with SAM (500 µM) in Tris buffer (pH 8.0) at 37 °C for indicated time (1, 3 or 5 hours), after which H3K9 (100 µM) was added and incubated for an additional hour. Unmodified (displayed as black bar), mono- (displayed as squares in green bar), di- (displayed as blue horizontal stripe blue bar) and tri- (displayed as pink vertical stripe in pink bar) represent the unmethylation and methylation states of the substrates and the products based on the mass peak integral at each time point.

**5. MALDI-TOF MS supplementary figures**


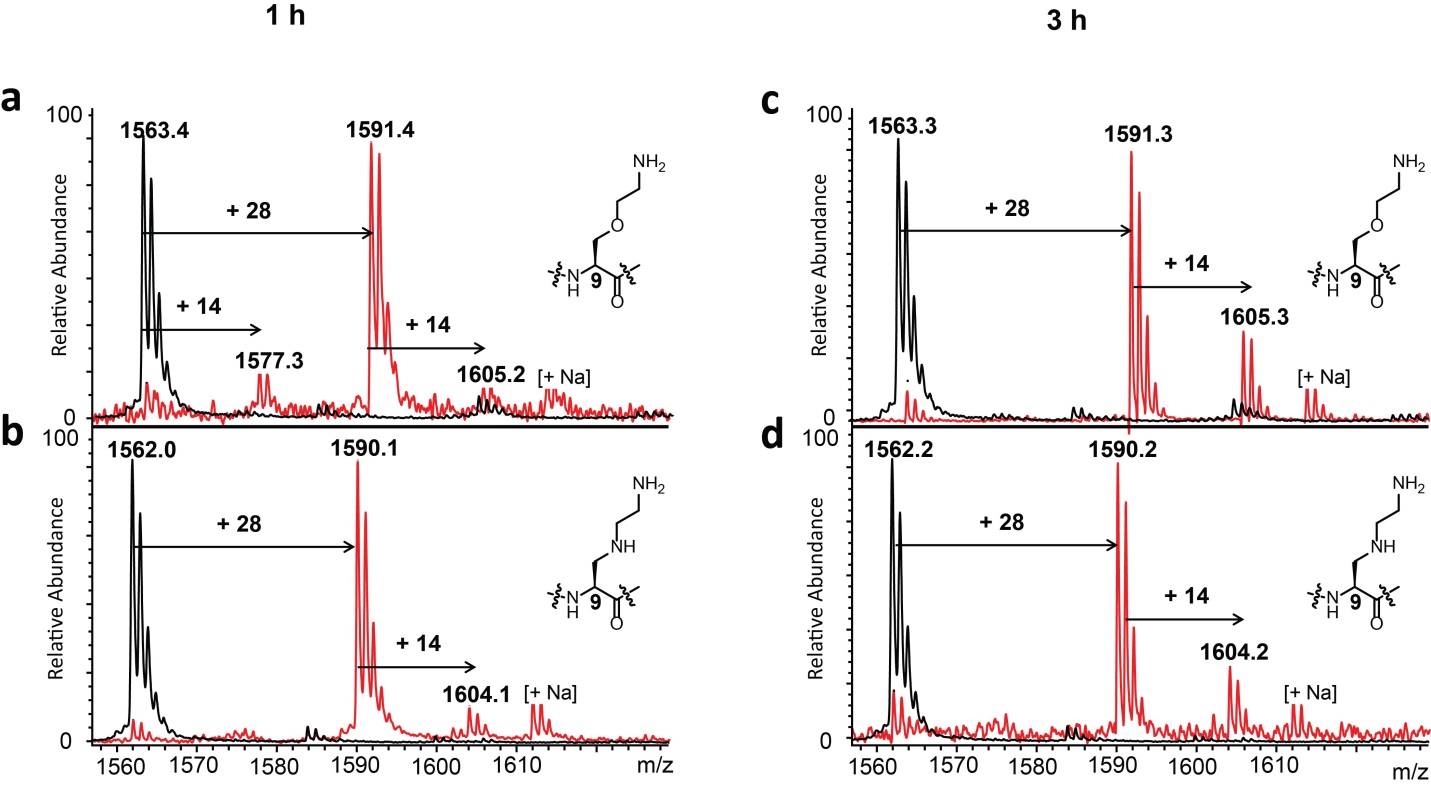


**Supplementary Figure 12.** MALDI-TOF MS analysis showing that GLP (10 µM) catalyzed methylation of all the two lysine analogues in the presence of SAM (1 mM) at 37 °C for 1 h (left panels) and longer incubation for 3 h (right panels). **a**) GLP catalyzed methylation of histone H3K_O_9 peptide to produce major dimethylation H3K_O_9me2 and species of monomethylation H3K_O_9me and traces of trimethylation H3K_O_9me3 for 1 h. **b**) GLP catalyzed methylation of histone H3K_N_9 peptide to produce major dimethylation H3K_N_9me2 and traces of trimethylation H3K_N_9me3 for 1 h. **c**) GLP catalyzed methylation of histone H3K_O_9 peptide to produce major dimethylation H3K_O_9me2 and species of trimethylation H3K_O_9me3 at longer incubation for 3 h. **d**) GLP catalyzed methylation of histone H3K_N_9 peptide to produce major dimethylation H3K_N_9me2 and species of trimethylation H3K_N_9me3 at longer incubation for 3 h. Red spectra show reactions including GLP enzyme and black spectra the no-enzyme controls.


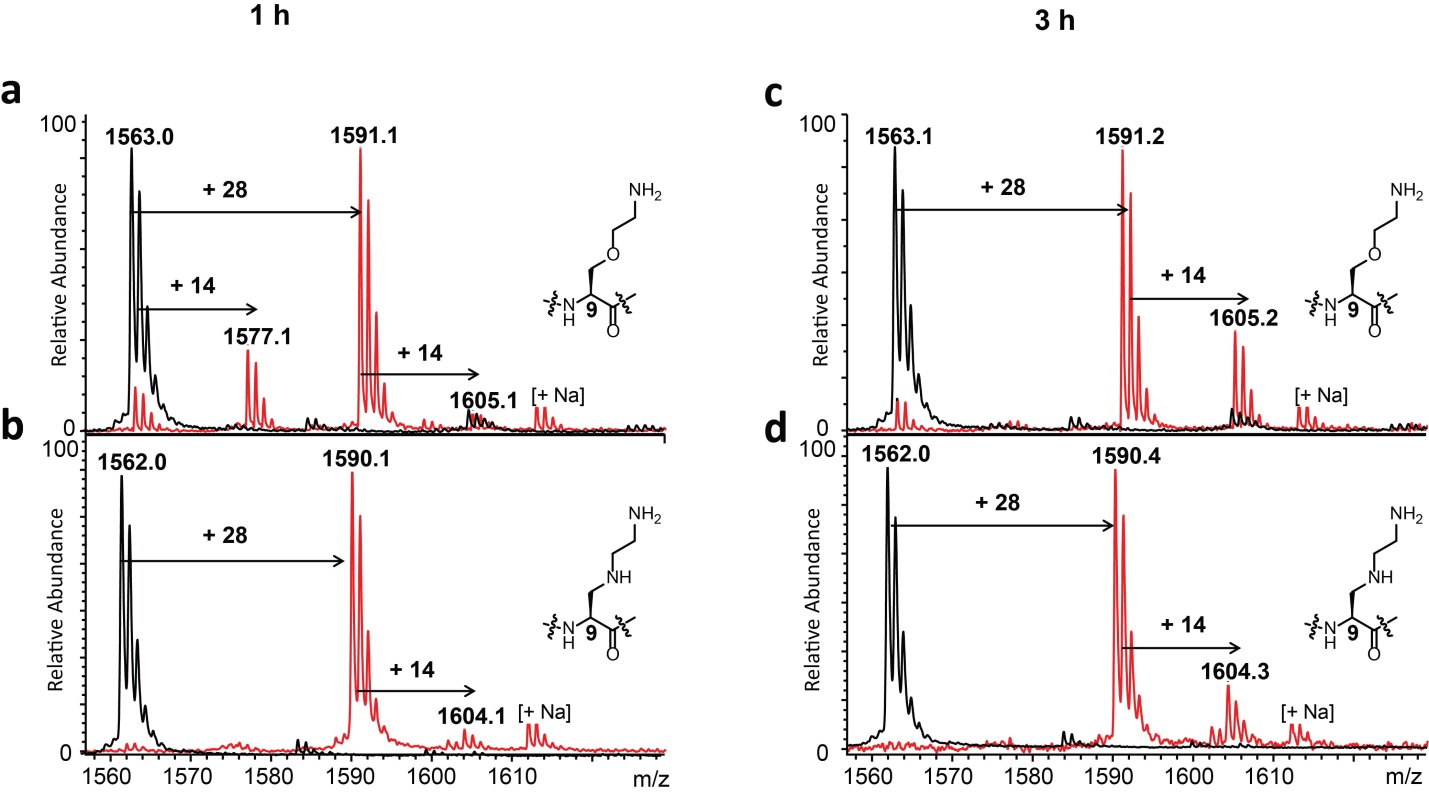


**Supplementary Figure 13.** MALDI-TOF MS analysis showing that G9a (10 µM) catalyzed methylation of all the two lysine analogues in the presence of SAM (1 mM) at 37 °C for 1 h (left panels) and longer incubation for 3 h (right panels). **a**) G9a catalyzed methylation of histone H3K_O_9 peptide to produce major dimethylation H3K_O_9me2 and species of monomethylation H3K_O_9me and traces of trimethylation H3K_O_9me3 for 1 h. **b**) G9a catalyzed methylation of histone H3K_N_9 peptide to produce major dimethylation H3K_N_9me2 and traces of trimethylation H3K_N_9me3 for 1 h. **c**) G9a catalyzed methylation of histone H3K_O_9 peptide to produce major dimethylation H3K_O_9me2 and species of trimethylation H3K_O_9me3 at longer incubation for 3 h. **d**) G9a catalyzed methylation of histone H3K_N_9 peptide to produce major dimethylation H3K_N_9me2 and species of trimethylation H3KN9me3 at longer incubation for 3 h. Red spectra show reactions including GLP enzyme and black spectra the no-enzyme controls.


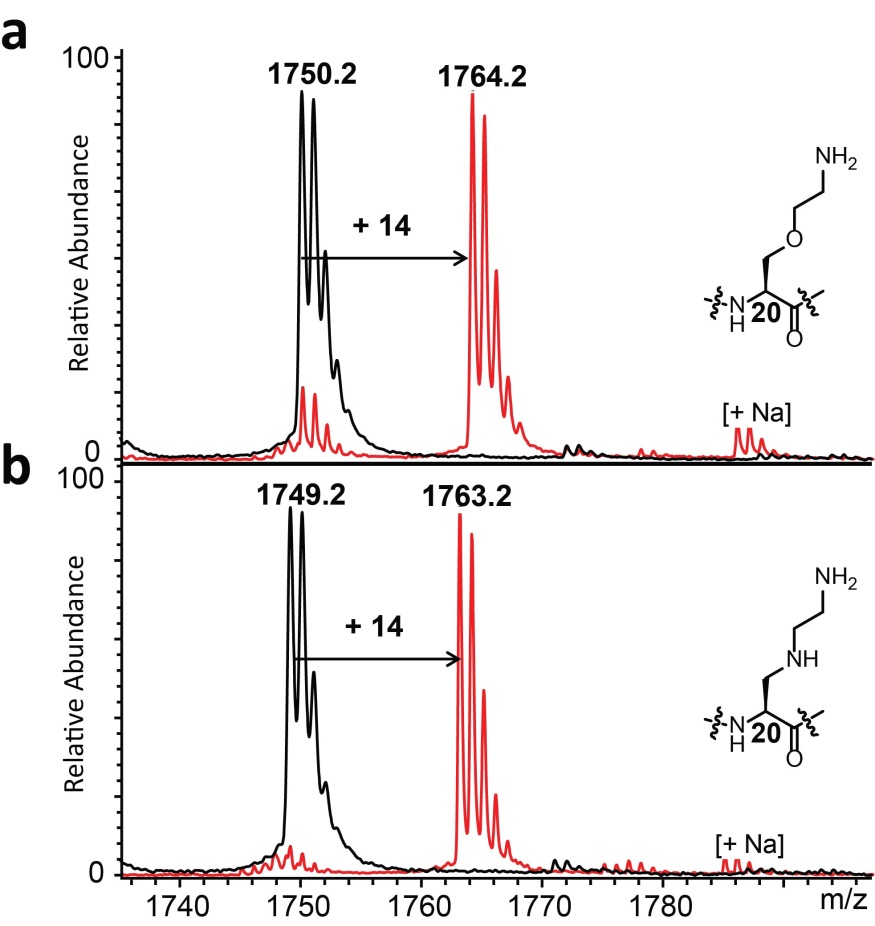


**Supplementary Figure 14.** MALDI-TOF MS analysis showing that SETD8 (10 µM) catalyzed methylation of the two lysine analogues in the presence of SAM (1 mM) at 37 °C for 1 h. **a**) SETD8 catalyzed almost full monomethylation of histone H4K_O_20 peptide to produce H3K_O_9me at 1 h. **b**) SETD8 catalyzed almost full monomethylation of histone H4K_N_20 peptide to produce H3K_N_9me at 1 h. Red spectra show reactions including SETD8 enzyme and black spectra the no-enzyme controls.


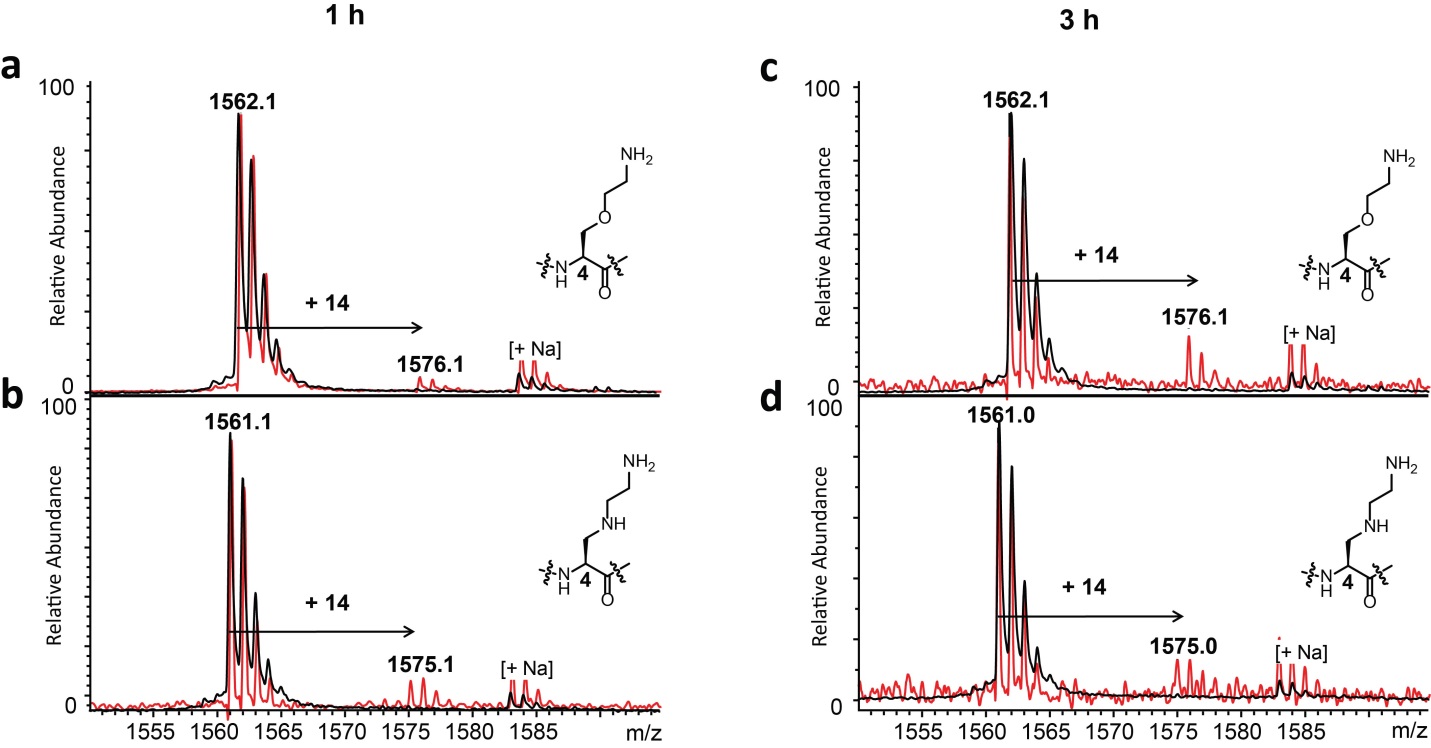


**Supplementary Figure 15.** MALDI-TOF MS analysis showing that SETD7 (10 µM) poorly catalyze methylation of the two lysine analogues in the presence of SAM (1 mM) at 37 °C for 1 h (left panels) and longer incubation for 3 h (right panels). SETD7 poorly catalyzed traces of histone H3K_O_4 peptide to produce H3K_O_4me at 1 h (**a**) and longer incubation for 3 h (**c**). SETD7 poorly catalyzed traces of histone H3K_N_4 peptide to produce H3K_N_4me at 1 h (**b**) and longer incubation for 3 h (**d**). Red spectra show reactions including SETD7 enzyme and black spectra the no-enzyme controls.

**6. Enzyme kinetics supplementary figures**


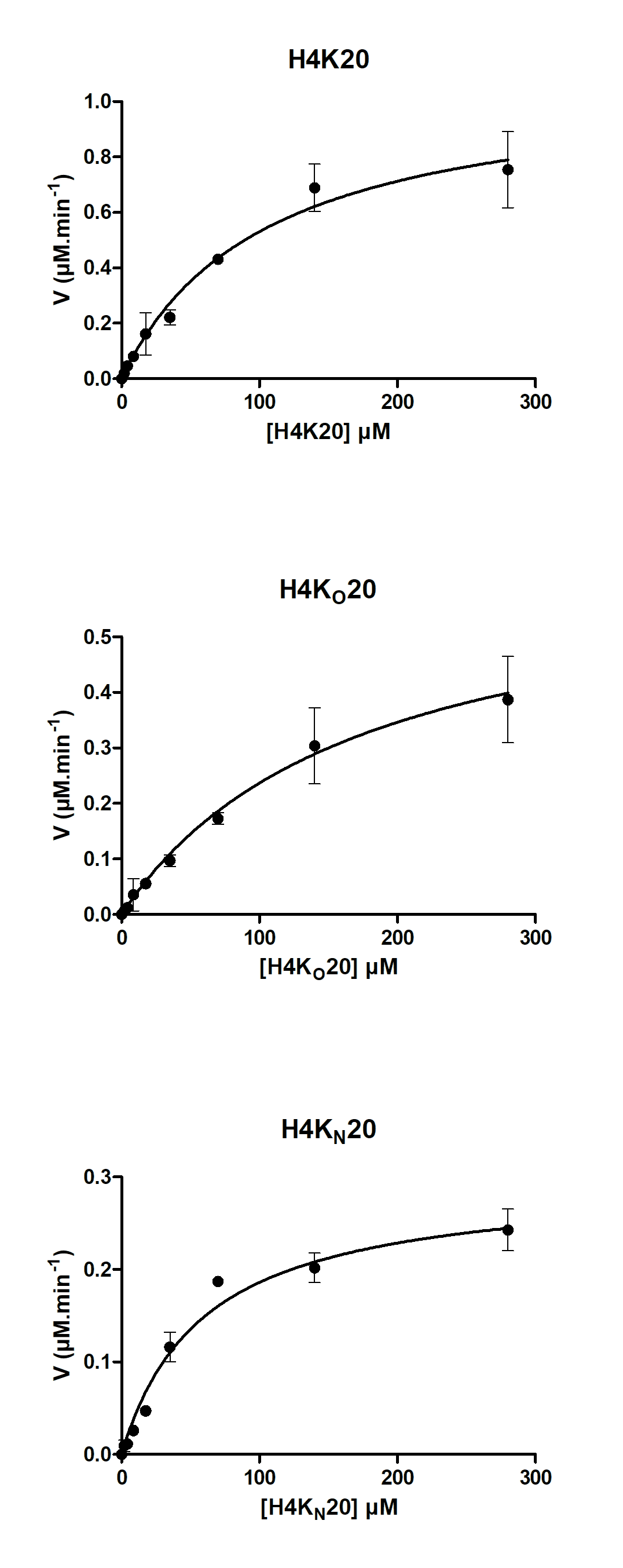


**Supplementary Figure 16.** Michaelis-Menten curves for reactions of the natural H4K20 peptide and lysine analogues H4K_O_20 and H4K_N_20 peptides with SETD8. Final values are reported as value ± SD.

**7. Inhibition supplementary figures**


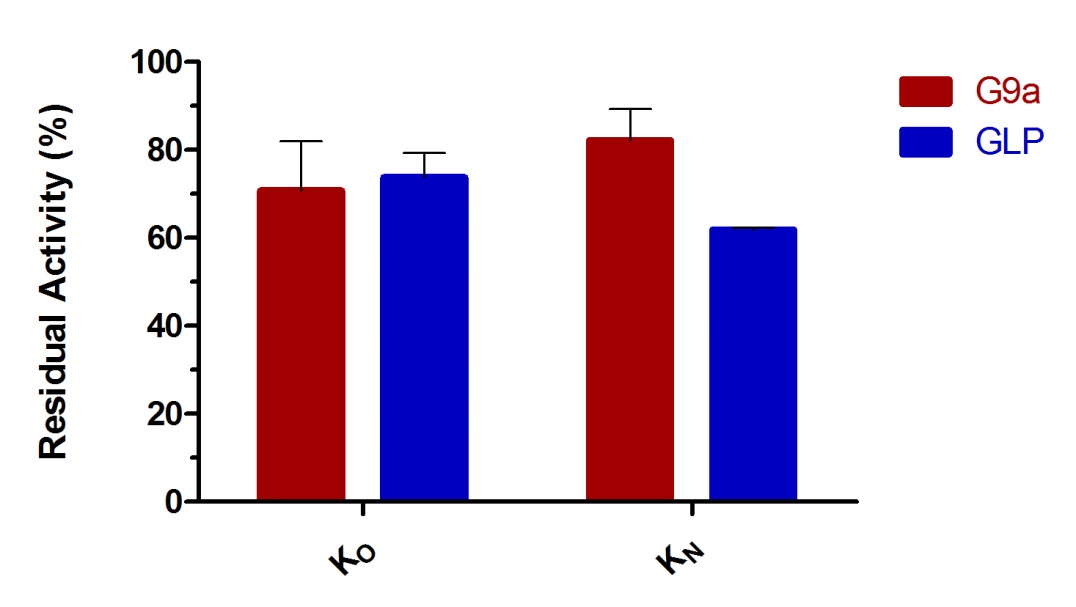


**Supplementary Figure 17.** Comparison of residual activity with the methyltransferases (100 nM) GLP (displayed in blue) and G9a (displayed in red) with 14-mer H3K9 (5 µM) and the γ-modified lysine analogues of H3K_O_9 and H3K_N_9 (100 µM) in 50 mM glycine pH 8.8 assay buffer for 30 min at 37 °C. Error bars denote the standard deviation of the mean

**
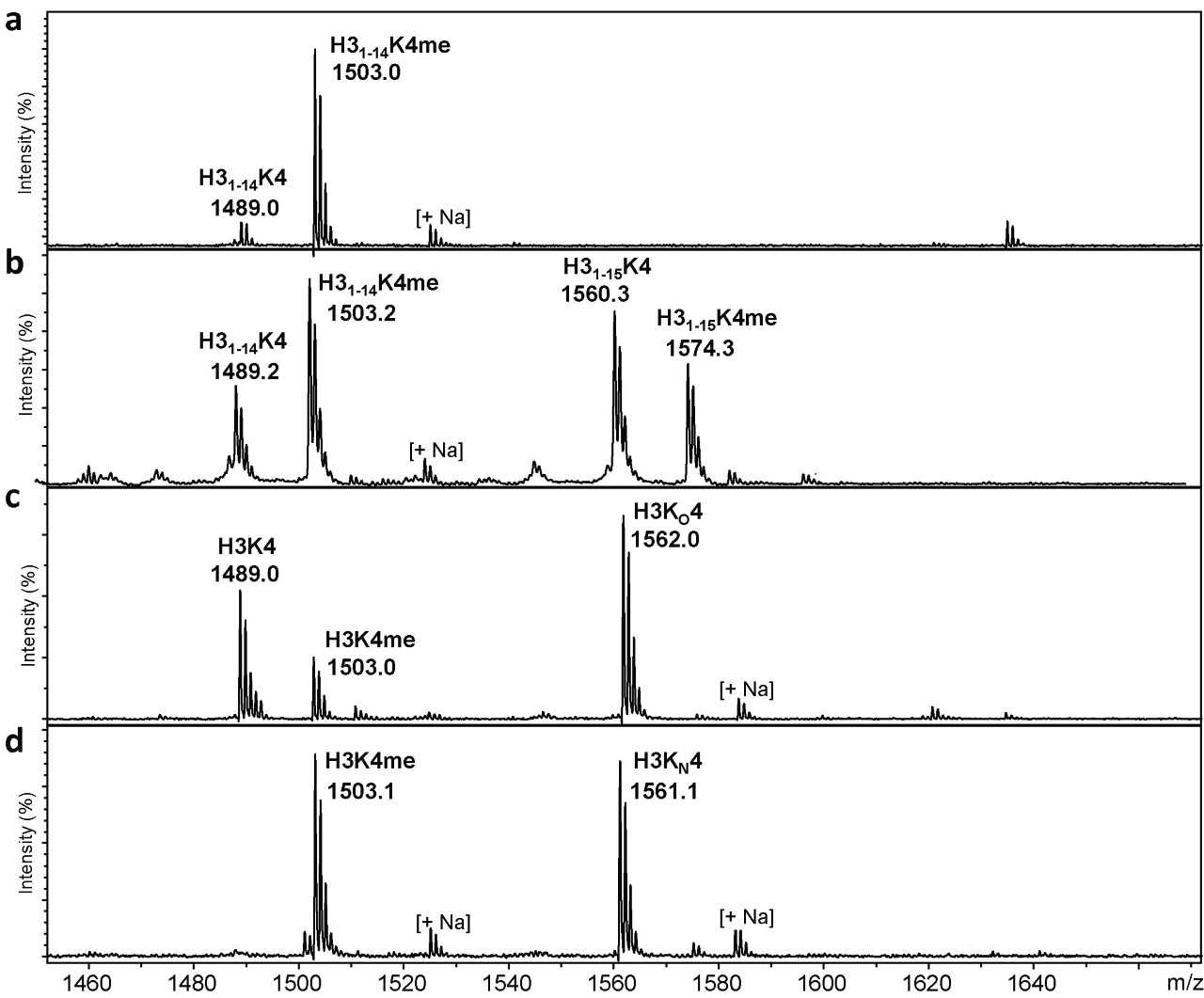
**

**Supplementary Figure 18**. MALDI-TOF MS based assay showing (a) SETD7-catalyzed monomethylation of H3_1-14_K4 (100 µM) in the presence of SAM (200 µM) after 1 h at 37 °C; (b) SETD7-catalyzed methylation of H3_1-14_K4 (100 µM) in the presence of H3_1-15_K4 (100 µM) and SAM (200 µM) after 1 h at 37 °C; (b) SETD7-catalyzed methylation of H3_1-14_K4 (100 µM) in the presence of H3K_O_4 (100 µM) and SAM (200 µM) after 1 h at 37 °C; (c) SETD7-catalyzed methylation of H3_1-14_K4 (100 µM) in the presence of H3K_N_4 (100 µM) and SAM (200 µM) after 1 h at 37 °C.

**

**

**Supplementary Figure 19.** Dose-response curve (IC_50_ = 38.9 µM) for inhibition of SETD7-catalyzed methylation (200 nM) of 14-mer H3K4 peptide (10 µM) by peptide inhibitor H3K_O_4 at various concentrations (1-200 µM). Error bars represent the standard deviation from 4 repeats.

**8. NMR supplementary figures**

**Supplementary Figure 20.** ^1^H NMR spectrum of the H3K_O_9 peptide (top). Multiplicity-edited HSQC data of the H3K_O_9 peptide (bottom; blue = positive, CH/CH_3_; red = negative, CH_2_).

**Supplementary Figure 21.** ^1^H NMR spectrum of the H3K_N_9 peptide (top). Multiplicity-edited HSQC data of the H3K_N_9 peptide (bottom; blue = positive, CH/CH_3_; red = negative, CH_2_).

**9. NMR spectra**

**
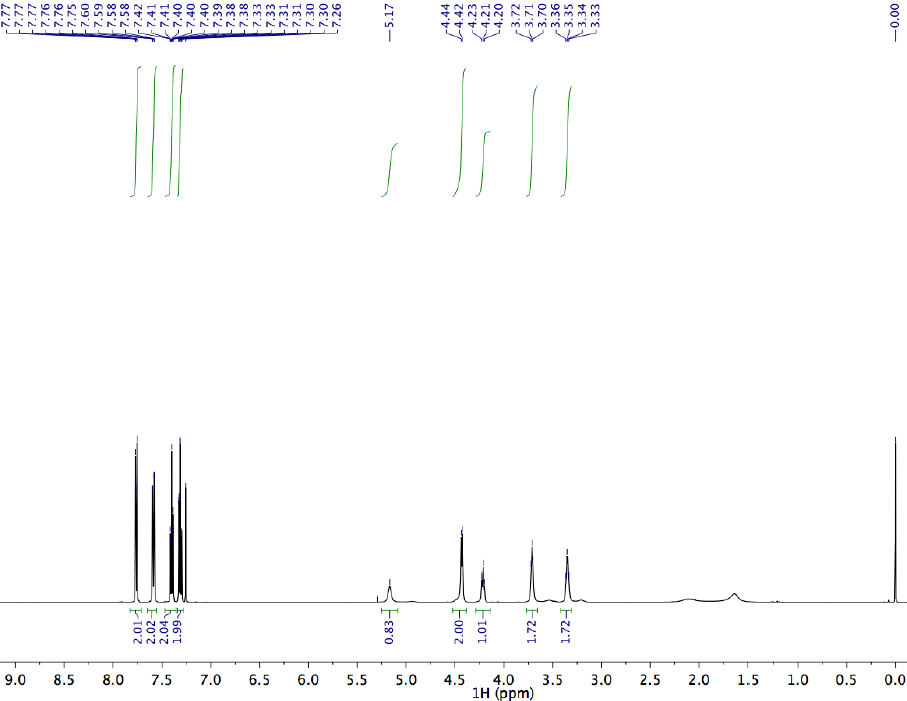
**

**
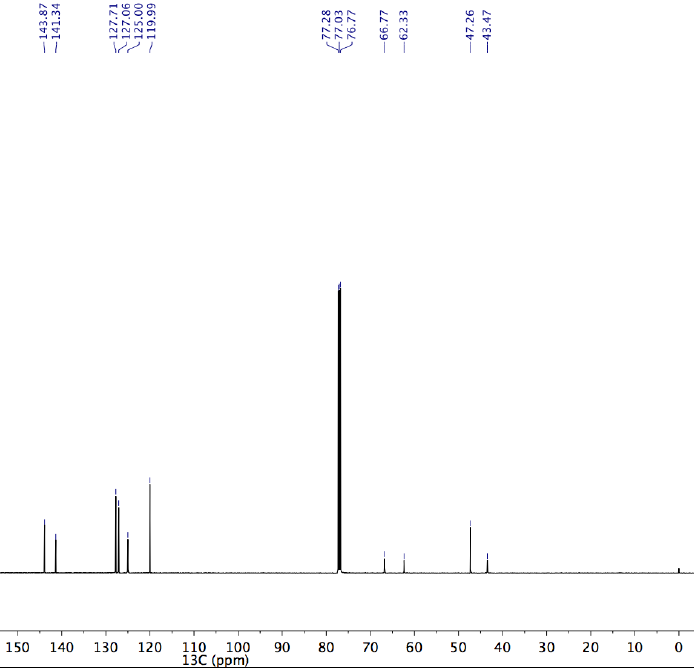
**

**
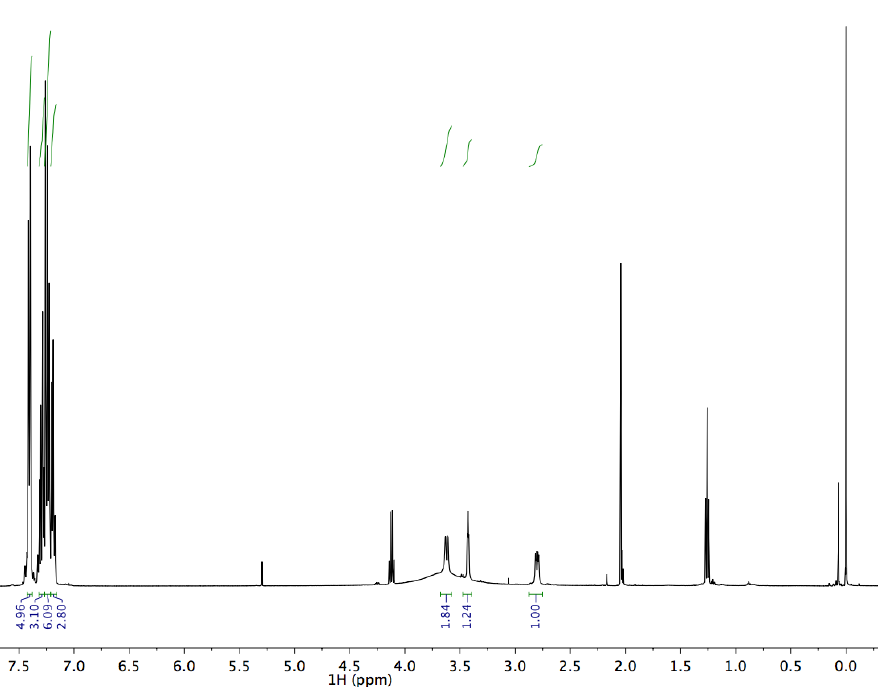
****
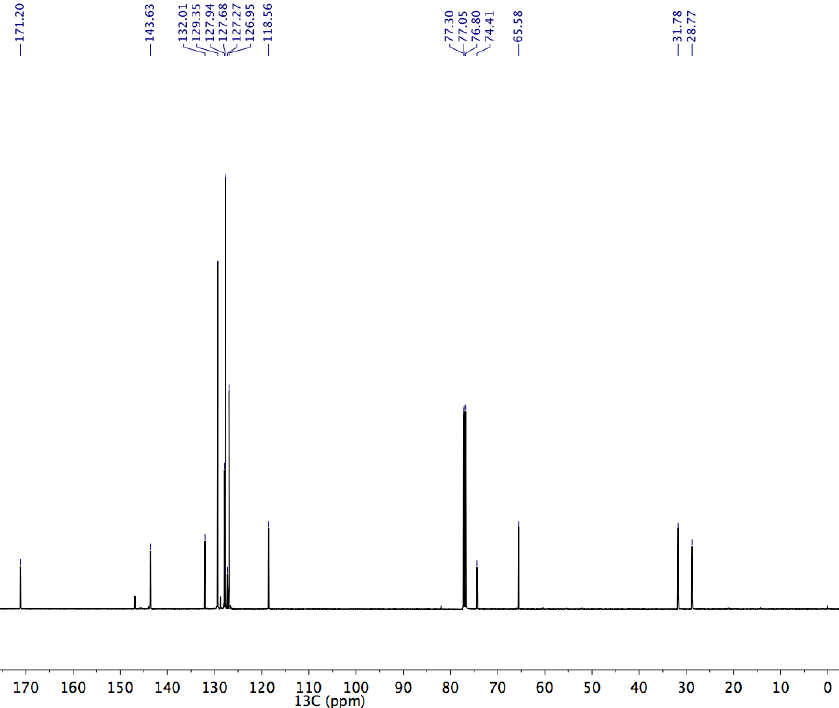
**

**
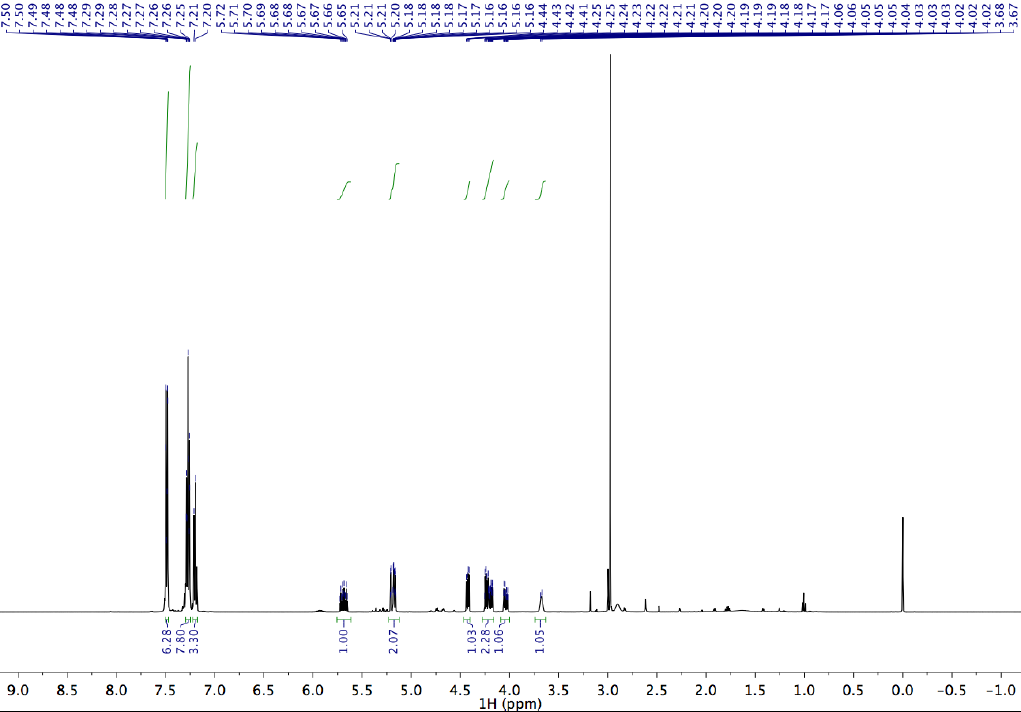

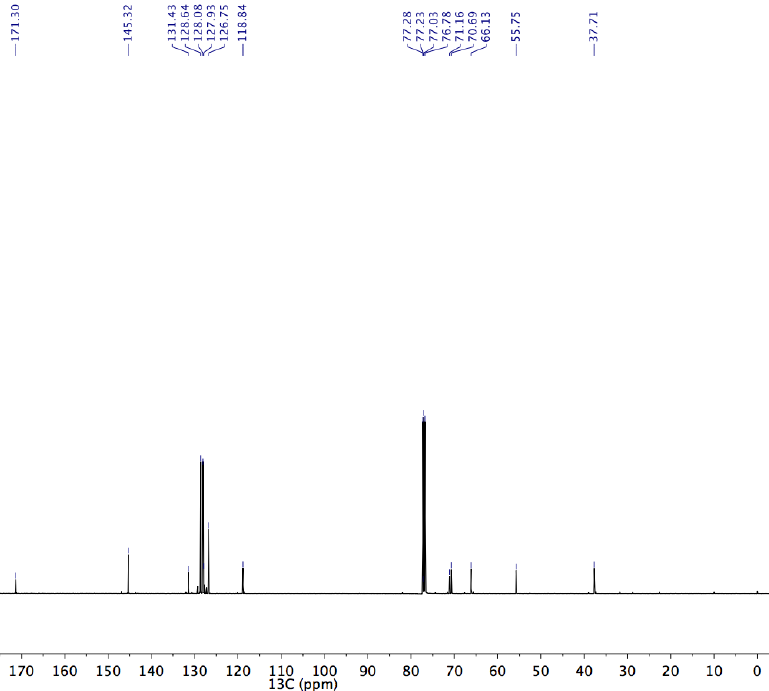
**

**
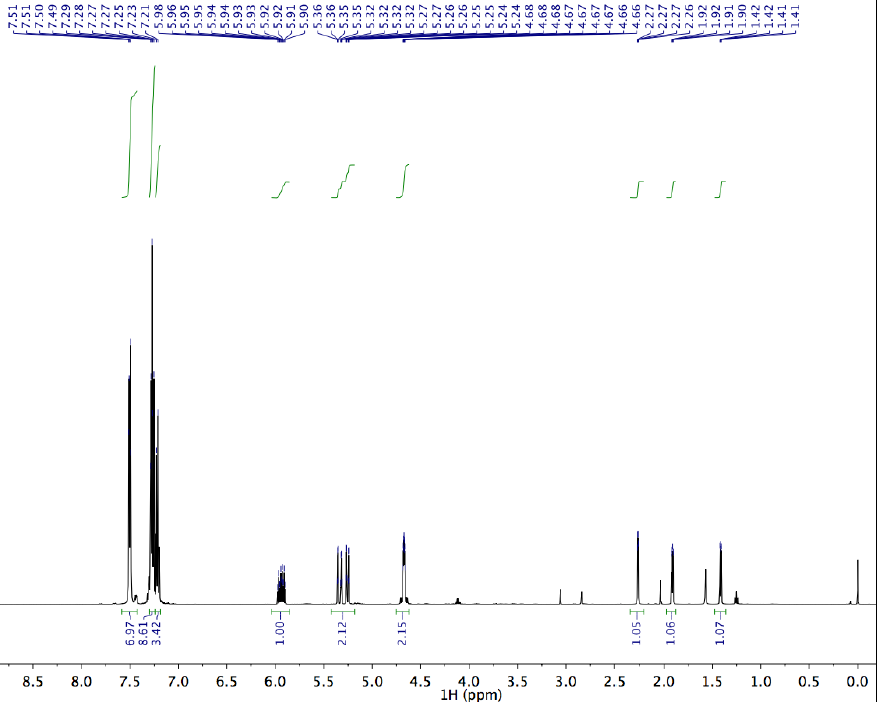
**

**
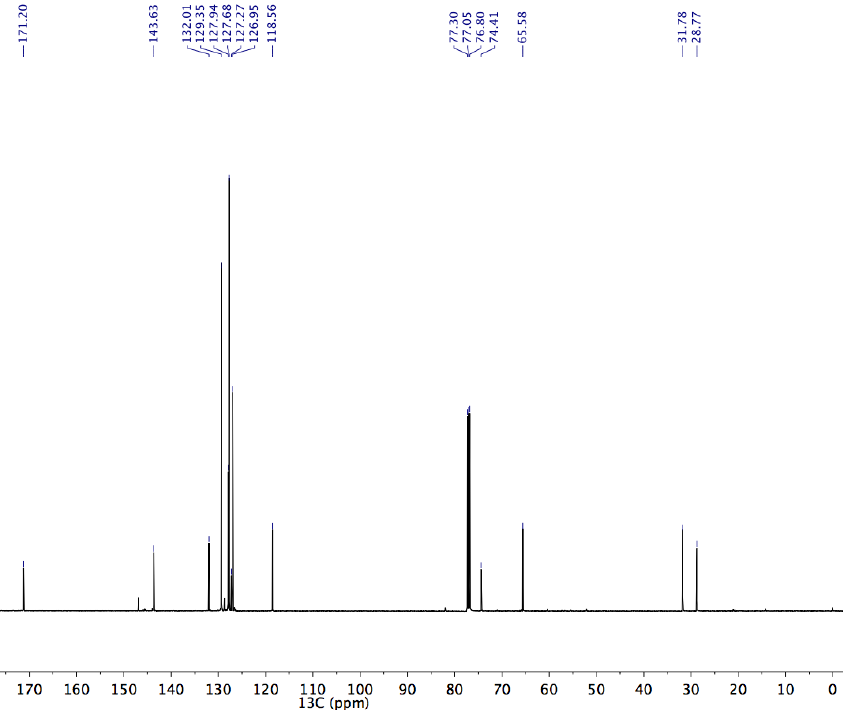
**

**
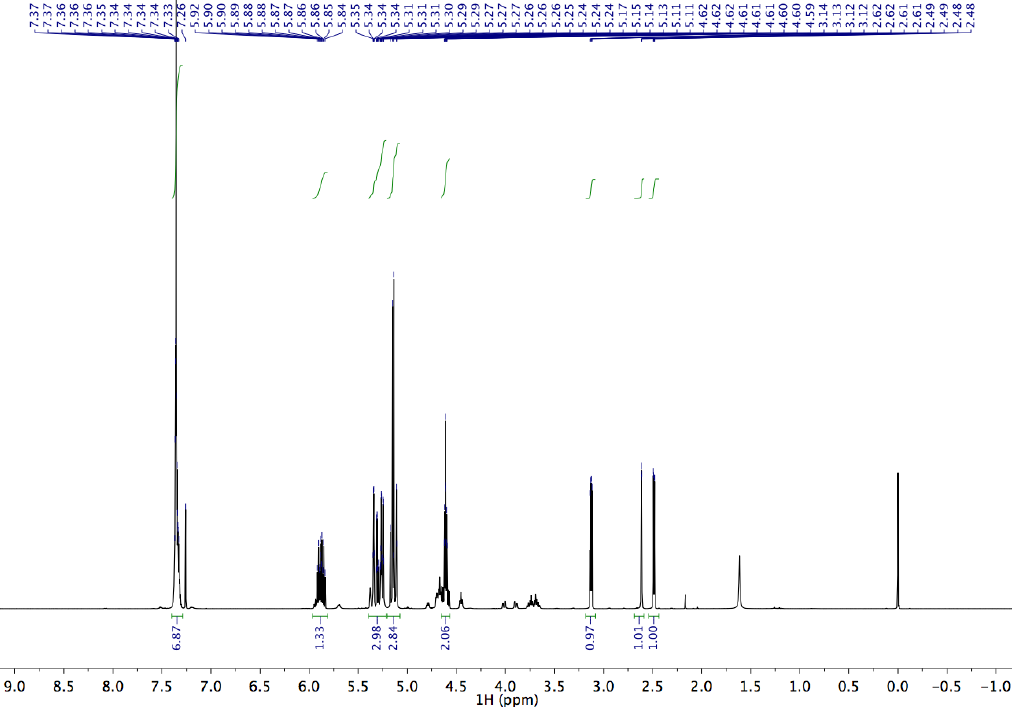
**

**
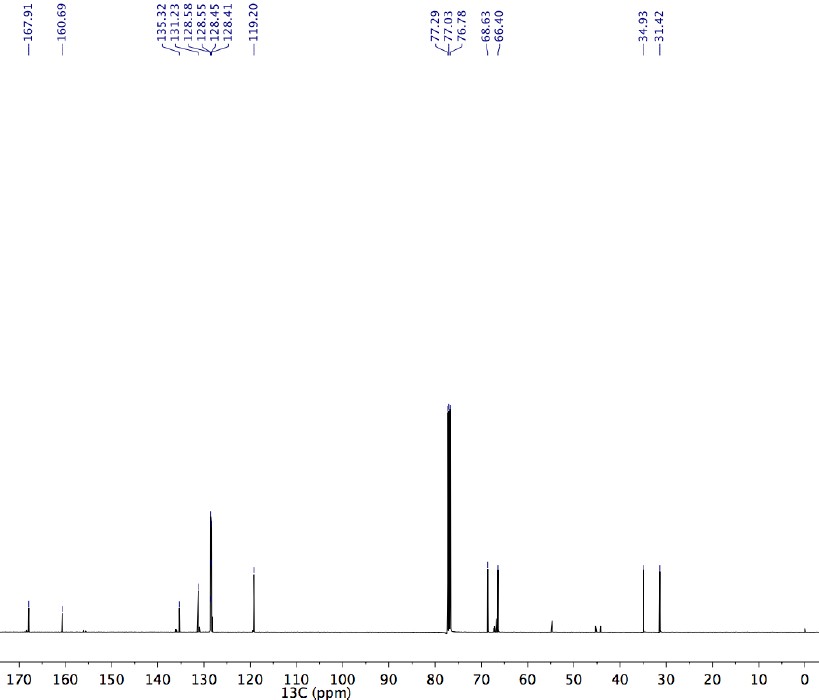
****
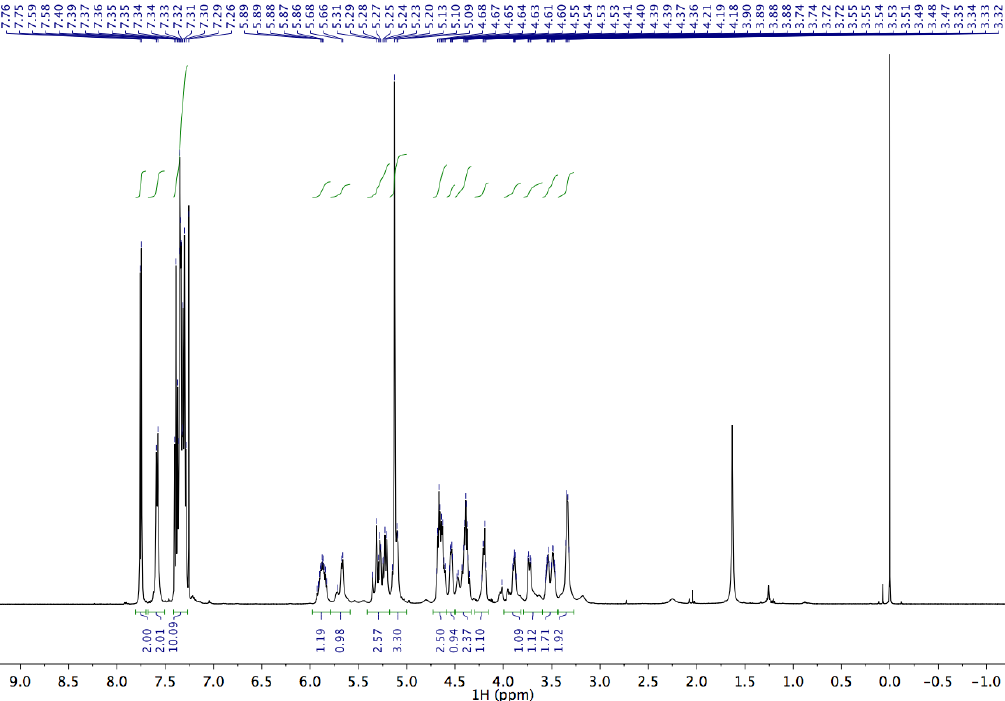
**

**
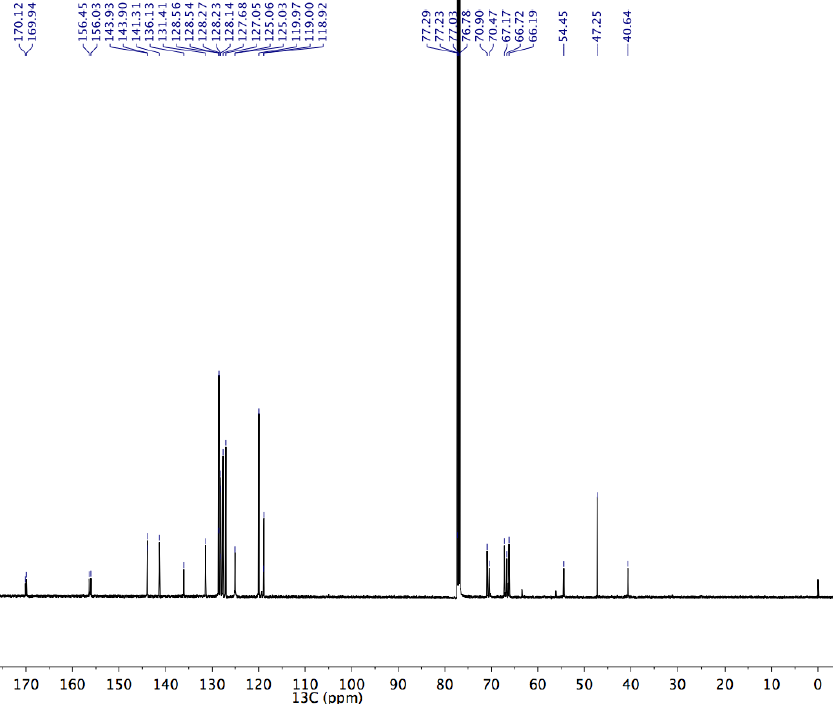
**

**
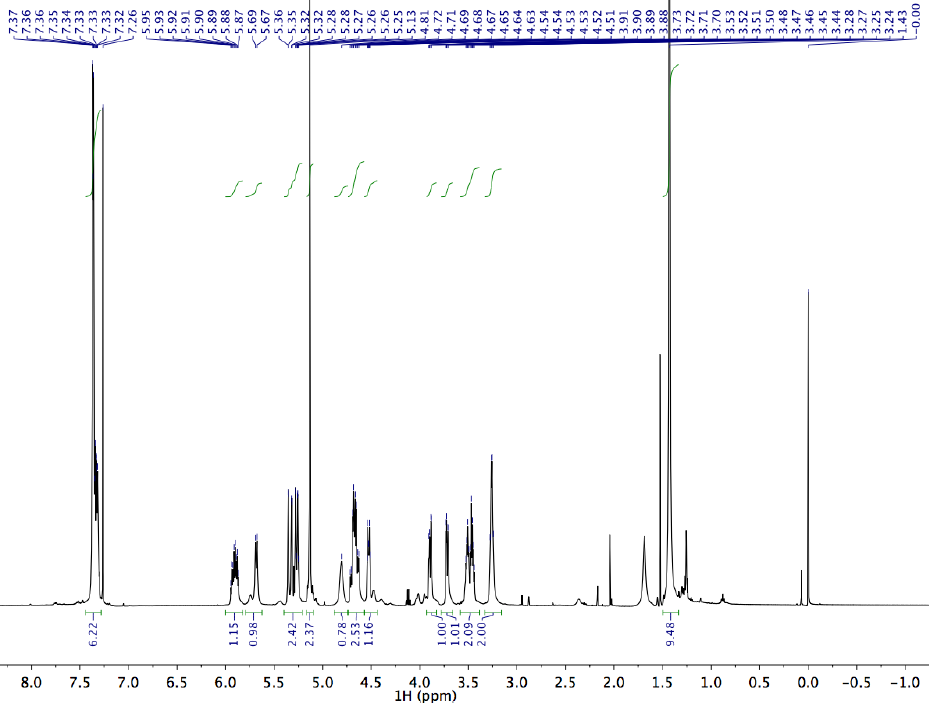
**

**
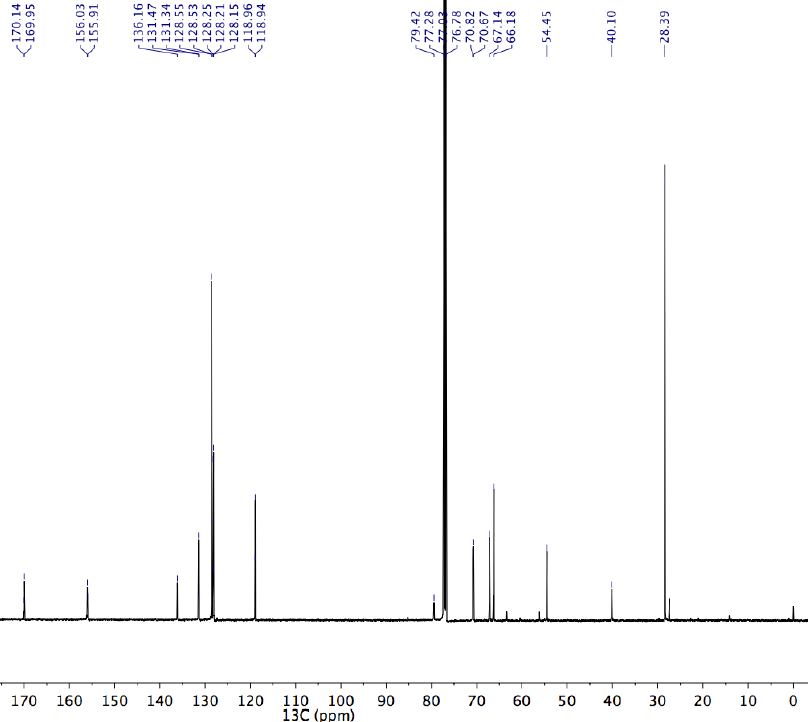
**
